# Neuronal traveling waves form preferred pathways using synaptic plasticity

**DOI:** 10.1007/s10827-024-00890-2

**Published:** 2024-12-27

**Authors:** Kendall Butler, Luis Cruz

**Affiliations:** Department of Physics, Drexel University, 3141 Chestnut Street, Philadelphia, 19104 PA USA

**Keywords:** Plasticity, Waves, Propagation, Learning

## Abstract

Traveling waves of neuronal spiking activity are commonly observed across the brain, but their intrinsic function is still a matter of investigation. Experiments suggest that they may be valuable in the consolidation of memory or learning, indicating that consideration of traveling waves in the presence of plasticity might be important. A possible outcome of this consideration is that the synaptic pathways, necessary for the propagation of these waves, will be modified by the waves themselves. This will create a feedback loop where both the traveling waves and the strengths of the available synaptic pathways will change. To computationally investigate this, we model a sheet of cortical tissue by considering a quasi two-dimensional network of model neurons locally connected with plastic synaptic weights using Spike-Timing Dependent Plasticity (STDP). By using different stimulation conditions (central, stochastic, and alternating stimulation), we demonstrate that starting from a random network, traveling waves with STDP will form and strengthen propagation pathways. With progressive formation of traveling waves, we observe increases in synaptic weight along the direction of wave propagation, increases in propagation speed when pathways are strengthened over time, and an increase in the local order of synaptic weights. We also present evidence that the interaction between traveling waves and plasticity can serve as a mechanism of network-wide competition between available pathways. With an improved understanding of the interactions between traveling waves and synaptic plasticity, we can approach a fuller understanding of mechanisms of learning, computation, and processing within the brain.

**Supplementary Information:**

The online version contains supplementary material available at 10.1007/s10827-024-00890-2.

## Introduction

Across the brain, and particularly in the neocortex, it has long been observed that neuronal activity often takes the form of propagating waves, which have been observed and studied in neurophysiological systems as early as 1934 (Adrian & Matthews, 1934). This spatial propagation of spiking activity across cortical networks has been observed in various *in vitro* models, which demonstrate the effect of specific neurotransmitters on wave velocity (Golomb & Amitai, 1997), as well as coupling between populations of cells affecting the shape and phase of observed waves (Bao & Wu, 2003). In disinhibited cortical slices, circular, spiral and irregular wave shapes can be observed (Huang et al., 2004), with some researchers investigating the distinct mechanisms of their initiation, propagation and termination in model tissues (Pinto et al., 2005). These data combined with the fact that traveling waves are commonly observed throughout the brain, suggest that the shape and direction of propagation may be relevant to their function.

Growing evidence supports the idea of various functional properties of traveling waves (Muller et al., 2018), including their potential importance in spatial processing (Benucci et al., 2007) and evidence of their role in feed-forward processing from higher to lower order sensory areas (Roland et al., 2006). In addition to sensory processing, other evidence suggests the importance of waves as a driving force for learning and refinement of neuronal circuits. A study on neonatal mice found that retinal waves propagate through the early visual system, demonstrating the potential importance of spontaneous traveling waves in the development of neuronal circuits (Ackman et al., 2012). Studies into cortical activity during sleep also demonstrate complex, slow-wave propagation patterns (Hangya et al., 2011), rotating sleep spindles (Muller et al., 2016), and the consistent propagation of slow-wave signals from the medial prefrontal cortex to the hippocampus and the medial temporal lobe (Nir et al., 2011). In the hippocampus, theta oscillations are observed to take the form of traveling waves (Lubenov & Siapas, 2009), moving along its septotemporal axis (Patel et al., 2012, 2013). Since both sleep states as well as hippocampal activity have been tied to the long-term consolidation of short-term memories, these results further suggest a role for traveling waves related to learning and memory processing.

Traveling waves will, naturally, travel along the synaptic paths that are available to them at the time of initiation. Neuronal pathways, which can be defined as the paths through which signals travel in the brain and the nervous system, have long been understood as important for a variety of processes, including visual processing (Madrid & Crognale, 2000), perception, action (Goodale & Milner, 1992), and even emotions as complex as empathy (Decety, 2015). Spontaneous wave propagation has also been observed as important in the development of early visual pathways (Shatz, 1996). Due to the importance of both traveling waves and the paths they travel through, it is important to investigate the extent to which propagating activity can form or strengthen these pathways in neuronal systems.

If traveling waves have a function in synaptic refinements and a role in pathway formation, they would require mechanisms of plasticity. Throughout life, many synapses learn as a result of the spike-timing correlations between presynaptic and postsynaptic cells (Zhang et al., 1998). One proposed and empirically supported rule for this type of learning is known as Spike-Timing Dependent Plasticity (STDP), which describes the strengthening or weakening of an input synapse to a postsynaptic cell (Song et al., 2000). STDP is a widely used learning rule in computational studies, and experiment has demonstrated that it, or similar biological plasticity rules, provide vital learning throughout the brain (Feldman, 2012). In sensory areas, STDP or STDP-like mechanisms have been studied and demonstrated to have importance in cortical organization during development as well as in the storage of sensory information later in life (Larsen et al., 2010). In rats, biological STDP has been observed and studied in the visual cortex (Meliza & Dan, 2006; Zilberter et al., 2009) the barrel cortex (Jacob et al., 2007) and somatosensory cortex (Letzkus et al., 2006). Similar observations of plasticity in the sensory areas have been achieved on mice (Banerjee et al., 2009) as well as on cats and humans (Yao & Dan, 2001; Schuett et al., 2001).

Because of the spatial overlap of the regions where traveling waves and STDP in the brain occur, it is important to investigate their interactions in biological networks to augment our understanding of their potential function. Previous work has in fact considered such interactions, such as using a reward-dependent STDP in a model network of Leaky integrate and fire (LIF) neurons (Ito & Toyoizumi, 2021), modeling path planning towards target locations (Ponulak & Hopfield, 2013), and variations in pattern formation as a function of wave propagation (Bennett & Bair, 2015). Further, noise enhanced signaling due to plasticity in an ‘STDP-driven network’ has also been demonstrated computationally (Lobov et al., 2017). A number of computational studies have also investigated traveling waves without the inclusion of synaptic adjustments, studying their dynamics (Gong & van Leeuwen, 2009; Keane & Gong, 2015; Paraskevov & Zendrikov, 2017), the meta-stability of spiral wave formations (Roberts et al., 2019) and potential mechanisms of navigational path planning (Khajeh-Alijani et al., 2015). While various studies have been completed, both in the form of observations as well as computational modeling of traveling waves, there is still missing important information on the direct interactions between STDP and the propagation of waves through neuronal tissues.

Here we present computational work that directly investigates pathway formation due to traveling waves using a theoretical model network with local connection probability and plasticity in the form of excitatory STDP. Our primary scientific question is whether traveling waves will form and stabilize propagation pathways, determined by synaptic weights, with the help of STDP. Our findings suggest that traveling waves form preferred pathways for the propagation of neuronal signals, increase the local order in the average direction of synaptic weight for nearest neighbor neurons, and increase propagation speed in the direction of formed pathways. In addition, our results support the idea that interactions between STDP and traveling waves can act as a mechanism of large-scale competition between available propagation pathways.

## Methods

### Computational setup

**Fig. 1:**
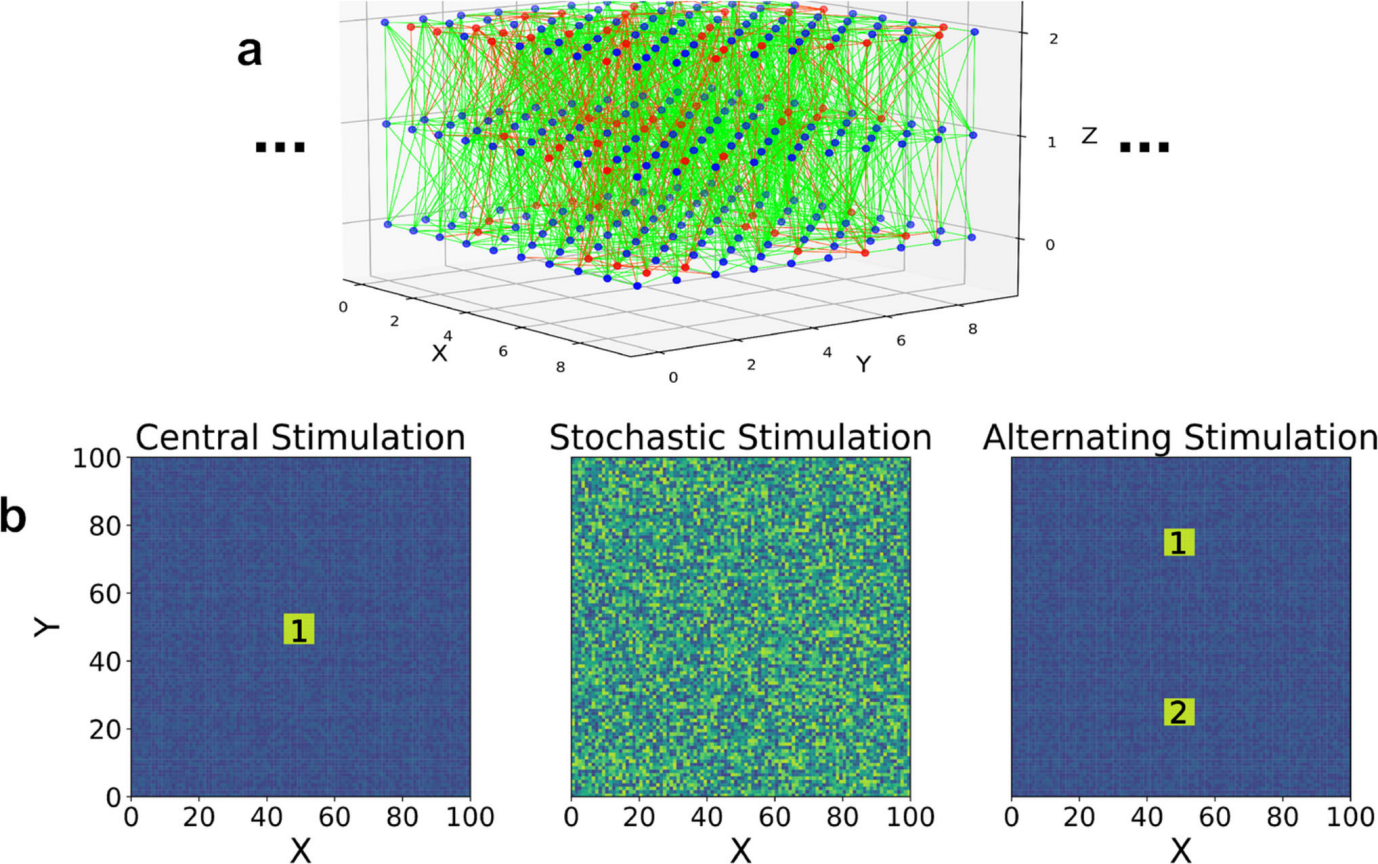
(a) 3D visualization of our network model only showing a $10 \times 10 \times 3$ section of the entire $100 \times 100 \times 3$ network. Blue dots are excitatory and red dots are inhibitory neurons, with accompanying synaptic connections that are green and red depending on whether they originate from excitatory or inhibitory neurons, respectively. (b) Visualizations of the three stimulation types. The first (left) is central stimulation, which consists of a 30 ms burst of stimulation at the highlighted site ‘1’ every 1000 ms. The second (middle) is stochastic stimulation, which consists of random, suprathreshold point stimulations distributed throughout the entire network. The third (right) is alternating stimulation, which consists of 30 ms bursts of stimulation alternating between the labeled sites ‘1’ and ‘2’ every 1000 ms

To study traveling waves in 2-dimensions, we construct a quasi-2D spiking network with random connections, local connection probability, and realistic spiking responses using the BRIAN neural simulator version 2.6.0 (Stimberg et al., 2019)^1^ This network is constructed by placing model neurons at 100 lattice positions along two long axes (*X*, *Y*) and 3 lattice positions along a short axis *Z*, shown in Fig. 1(a). Positions along each axis begin at 0, so the range of neuron positions in (*X*, *Y*) are from 0 to 99 and positions in *Z* are from 0 to 2. The inclusion of 3 positions in Z was chosen phenomenologically, as we found that the additional network layers allow for more robust wave propagation, whereas equivalent experiments with a single layer yielded little to no wave propagation (Baker, 2022). Each neuron is randomly chosen as excitatory or inhibitory with a probability that resulted in 80% excitatory and 20% inhibitory, reflecting commonly observed population percentages across species (Hendry et al., 1987). Connectivity between neurons is modeled by a random distribution dependent on the distance between neurons, given by (Maass et al., 2002),

1 $$\begin{aligned} P_{ij} = C e^{-(\frac{D(i,j)}{\lambda })^2} \end{aligned}$$

where $P_{ij}$ is the connection probability between pre and postsynaptic neurons *i* and *j*, *C* is a constant probability coefficient, *D*(*i*, *j*) is the Euclidean distance between neurons, and $\lambda$ is a characteristic connectivity length of the network. The values of *C* and $\lambda$ are taken to be 0.6 and 2.5, respectively, following previous work (Baker & Cruz, 2021). This distribution has been observed in the mouse auditory cortex (Levy & Reyes, 2012). For this connectivity scheme, multiple connections between the same pre and postsynaptic neurons are prohibited while mutual single connections between two neurons are not. Autapses, or synaptic connections between a neuron and itself, are also not included in our model. Additionally, the network is considered to have open boundary conditions at its edges.

The firing dynamics of each neuron is modeled by using the Izhikevich model (Izhikevich, 2003), which consists of two coupled differential equations,

2 $$\begin{aligned}&\frac{dv}{dt} = 0.04 v(t)^2 + 5v(t) + 140 - u(t) + I \end{aligned}$$

3 $$\begin{aligned}&\frac{du}{dt} = a(bv(t)-u(t)) \end{aligned}$$

4 $$\begin{aligned}&\text {if} \; v>30 \; ; \; v \leftarrow c \; , \; u \leftarrow u + d \end{aligned}$$

where *v* is a unit-less representation of the membrane potential, *u* is the membrane recovery variable, *I* is the sum of input currents from internal synapses as well as external inputs, and variables *a*, *b*, *c* and *d* are adjustable parameters that modulate the spiking activity of the neuron. Equation (4) describes a spike, which occurs when *v* surpasses a value of 30, causing a reset of *v* to the value of *c* and an increment of *u* by the value of *d*. The ranges of values of *a*, *b*, *c* and *d* used here are listed in Table 1 and were taken from Izhikevich (2003) that allow model neurons to recreate a wide diversity of observed cortical firing responses for excitatory and inhibitory neurons, including but not limited to regular spiking, fast spiking, and intrinsically bursting spike patterns.

**Table 1:** Neuron Parameters

Parameter	Excitatory	Inhibitory
a	0.02	[0.02, 0.1]
b	0.2	[0.2, 0.25]
c	[-65, -55]	-65
d	[2, 8]	2

Implemented values for the parameters of the Izhikevich model neurons used here. [A,B] represents a value taken from a uniform random distribution between *A* and *B*

One vital property of neuronal populations in relation to traveling waves is the time delay between the presynaptic spike and the arrival of that signal to the postsynaptic neuron (Katz & Miledi, 1965). We model these time delays as distance dependent by the following equations,

5 $$\begin{aligned}&\tau _{ij} = \kappa D(i,j) \end{aligned}$$

6 $$\begin{aligned}&\text {if} \;\; t = t_i' + \tau _{ij} \; ; \; I_j \leftarrow I_j + W_{ij} \end{aligned}$$

7 $$\begin{aligned}&\frac{dI_j}{dt} = -\frac{I_j}{\sigma _s} \end{aligned}$$

where $\tau _{ij}$ is the time delay for signals traveling from neuron *i* to neuron *j*, $\kappa$ is a constant that adjusts the scale of delay times here set to 0.5, *D*(*i*, *j*) is the Euclidean distance between pre and postsynaptic neurons, $t_{i}^{'}$ is the time at which a presynaptic spike occurs, $I_j$ is the total input current into the postsynaptic neuron, $W_{ij}$ is the synaptic weight of the connection from neuron *i* to *j*, and $\sigma _s$ is the characteristic time for the total input current set to $4\, ms$, a value within the range of synaptic response times for real neurons (Clements et al., 1992; Clements, 1996). Equation (6) indicates that at a time $\tau _{ij}$ after the presynaptic neuron fires at $t_{i}^{'}$, the postsynaptic current $I_j$ is incremented by the value of the weight $W_{ij}$. Equation (7) prescribes that the total input current decays exponentially over time with a characteristic time of $\sigma _s$.

The initial values of all synaptic weights are determined for both inhibitory and excitatory synapses according to,

8 $$\begin{aligned}&W_{ij}^{exc} = K \cdot U(0,0.5) \end{aligned}$$

9 $$\begin{aligned}&W_{ij}^{inh} = -K \cdot U(0,1) \end{aligned}$$

where $W_{ij}^{exc}$ and $W_{ij}^{inh}$ are the synaptic weights between neurons *i* and *j* for excitatory or inhibitory synapses, respectively. *K* is a parameter that adjusts the initial range of the synaptic weights, here taken as 11.0, and *U*(*A*, *B*) is a value taken from a uniform random distribution between *A* and *B*.

All external input into the network is implemented by stochastic Poisson point processes injected as spike trains into individual neurons. Unless specified otherwise, the magnitude of the input currents are given by the following equations,

10 $$\begin{aligned}&(I_{i}^{exc})_s = M \cdot U(0,1) \end{aligned}$$

11 $$\begin{aligned}&(I_{i}^{inh})_s = \frac{2}{5} M \cdot U(0,1) \end{aligned}$$

where $(I_{i}^{exc})_s$ and $(I_{i}^{inh})_s$ are input currents into the excitatory or inhibitory neuron *i*, respectively, for each input spike *s*. The constant *M* adjusts the range of the input values. The stochastic point processes are generated at an average frequency $\nu$. Note that these input spikes will correspondingly increase the total current *I* given in Eq. (6).

We include synaptic plasticity by implementing STDP (Zhang et al., 1998; Song et al., 2000) to excitatory synapses, in which the time-difference between pre and postsynaptic spikes directly affect whether synaptic efficacy is increased (long-term potentiation, LTP) or decreased (long-term depression, LTD). The equations describing this process are given by,

12 $$\begin{aligned} \Delta W_{ij}^{exc}= &  {\left\{ \begin{array}{ll} +R\cdot a_+\cdot e^{-\frac{|\Delta t|}{\tau _+}} & \text{ if } \Delta t < 0 \\ - R\cdot a_- \cdot e^{-\frac{|\Delta t|}{\tau _-}} & \text{ if } \Delta t>0 \end{array}\right. } \end{aligned}$$

13 $$\begin{aligned} \Delta t= &  t_i - t_j \end{aligned}$$

where $\Delta W_{ij}^{exc}$ is the change in synaptic weight between neurons *i* and *j* for a pair of presynaptic and postsynaptic spikes, *R* is an independent variable that we use to parameterize the scale of weight changes. The coefficients $a_+$ and $a_-$ are coefficients controlling the scale of individual weight changes, $\Delta t$ is the time difference between the postsynaptic spike $t_i$ and the presynaptic spike $t_j$, and $\tau _+$ and $\tau _-$ are the exponential time constants for LTP and LTD, respectively. Both $a_+$ and $a_-$ are set equal to 0.0016, and the time constants are taken to be asymmetric, with $\tau _- = 32 \; ms$ and $\tau _+ = 16 \; ms$ that correspond to observed values (Song et al., 2000; Feldman, 2000). The independent parameter *R* is used to investigate whether the scale of weight changes for individual pairs of spikes significantly impact the response of the network, and included values are $R = 0, 1, 2, 3,$ and 4. We note that we restrict weights to the initial maximum and minimum values equal to $0.5 \cdot K$ and 0 respectively (Eq. (8)) to avoid divergence of synaptic weights towards increasingly larger values.

These weight changes are algorithmically implemented using pre and postsynaptic trace variables that are incremented by the value of the coefficients ($R\cdot a_+$/$-R \cdot a_-$) after pre and postsynaptic spikes respectively and decay continuously as described in the above equation. When the opposite neuron fires, the synaptic weight is adjusted by the current value of the corresponding trace variable. In this way, all pairs of pre and postsynaptic neuronal spikes are included when calculating changes in weights.

All averages of the quantities that characterize the networks (described below) are obtained by generating and averaging simulations using 40 random seeds. The value of a random seed affects all randomized values and properties of the system, including the internal parameters of each neuron, the distribution of inhibitory and excitatory neurons, whether any two neurons are connected via a synapse, the initial weight of synapses, the timing of stochastic input spikes and the resultant input current of said spikes, thus making each system and stochastic inputs fully unique for each individual random seed. Integration of the differential equations is computed using the Euler method with a timestep of $0.1\, ms$. Additional results and figures not presented in the main body of the manuscript are presented in the Supplementary Information and are labeled with an ‘SI’.

### Input types

To address the overarching question of the role of STDP in pathway formation by traveling waves, we investigate three specific questions using numerical experiments that differ in input stimulation, schematized in Fig. 1(b). The first determines whether traveling waves form and strengthen pathways in a predictable way. For this we use a **central stimulation** consisting of a burst of supra-threshold stimulation occurring at the center of the network in a $8\times 8$ region centered at $\langle 49.5,49.5 \rangle$ with a constant input magnitude of $I_{in} = 4$ and a poisson frequency of $\nu = 500$ Hz. This burst lasts for $30\, ms$ and is repeated every $1,000\, ms$. We also include sub-threshold background noise across the entire network with inputs as described by Eqs. (10-11), with $M = 0.5$ and $\nu = 100$ Hz. The goal of this input category is to initiate a circular traveling wave that propagates outward from the center of the network at regular intervals. With predictable outward propagation, this experiment examines the outcome when the same pathways are repeatedly and predictably activated.

The second experiment tests whether the formation of pathways through networks is robust regardless of the shape, location, and direction of the wave propagation. For this we use a **stochastic stimulation** that consists of supra-threshold inputs across the entire network. The inputs are single Poisson point processes per neuron with input spikes described by Eqs. (10-11) with $M = 1.8$ and a Poisson frequency (average spike frequency) of $\nu = 180$ Hz. These values for $\nu$ and *M* are chosen such that they are large enough to produce well-formed traveling waves across the network while still being within biological ranges for the frequency of thalamic inputs (McCormick & Bal, 1997; Steriade, 2000). Because waves are not originating from predictable locations as in the central stimulation above, this experiment investigates whether pathways are formed and stabilized over time even under unpredictable wave propagation.

The third experiment investigates the coexistence of pathways created by different traveling waves. For this we use an **alternating stimulation** that consists of alternating external bursts of stimulation at different locations in the network. The alternating bursts occur at two $8\times 8$ regions centered at $\langle 49.5,74.5 \rangle$ (top) and $\langle 49.5, 24.5 \rangle$ (bottom) of the network, both receiving equivalent stimulation to that described for the central stimulation above, and alternating every $1,000 \, ms$. As above, we include a whole-network sub-threshold background noise stimulation with $M = 0.5$ and $\nu = 100$ Hz. Because the region between the two centers of stimulation receive temporally alternating waves propagating in opposite directions every $1000\, ms$, this experiment quantifies any long-term effects that competing pathway formation may have on the network.

### Metrics of pathway formation

To quantify and visualize the characteristics and evolution of pathway formation, we construct several metrics described below. As the basic concept characterizing pathways is the consideration of excitatory synapses as vectors, $\vec {W_{ij}}$, with a magnitude equal to the value of the weight $W_{ij}$ and direction defined by the orientation within our lattice of a vector originating at the excitatory presynaptic neuron *i* and ending at the postsynaptic neuron *j*. With this definition, we can readily quantify regional changes in the synaptic weights from the start to the end of a selected period $\Delta t_{w}$ by defining the average weight change vector $\Delta \vec {W}_s(\Delta t_{w})$ in region *s* by,

14 $$\begin{aligned} \Delta \vec {W}_s(\Delta t_{w}) = \frac{1}{{N_{syn}}}\sum _{i',j'}^s \Delta \vec {W_{i'j'}}(\Delta t_{w}) \end{aligned}$$

where $\Delta \vec {W}_{i'j'}(\Delta t_{w})$ is the change in synaptic weight between neurons $i'$ and $j'$ over the same time period, and $N_{syn}$ is the total number of excitatory synapses in the corresponding region *s*.

**Fig. 2:**
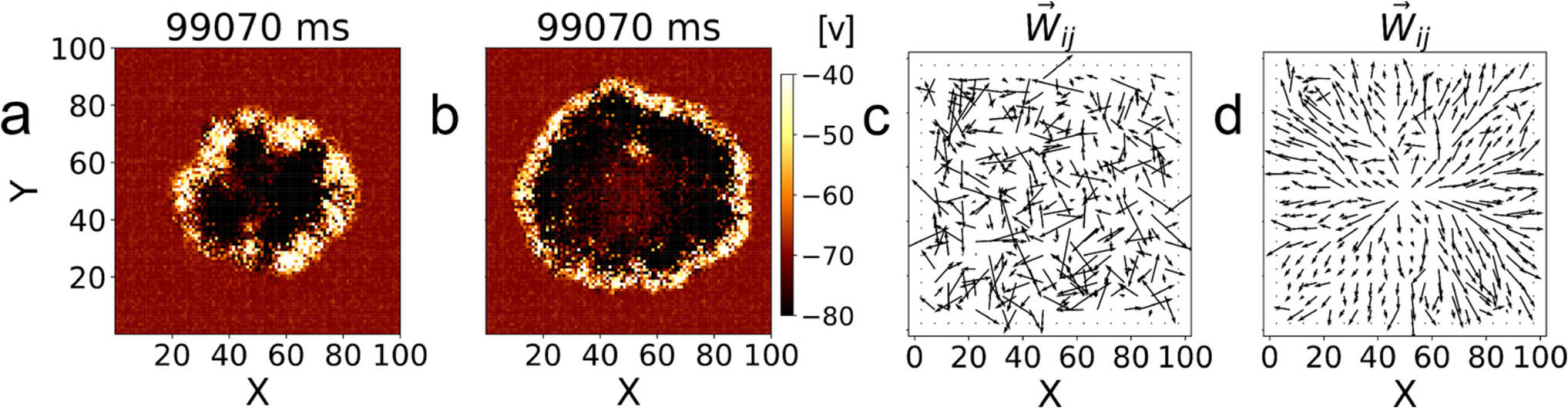
Comparison of the network activity and synaptic weights with and without plasticity. (a) shows network activity 70 ms after the $i=100$ stimulation on a network without synaptic plasticity ($R = 0$). (b) shows network activity 70 ms after the $i=100$ stimulation on a network with synaptic plasticity ($R=4$). The color of each (*X*, *Y*) position represents the average membrane potential *v* of the three lattice positions along the *Z* direction. Vector field of the average synaptic weights for each $5\times 5$ region of the networks (excluding edge regions) after the $i=100$ stimulation without STDP and with ($R=4$) STDP are shown in (c) and (d), respectively

To measure orientational order, we define an average local order parameter *O* that measures the degree of orientation between adjacent weight vectors and is given by,

15 $$\begin{aligned} O = \left\langle \frac{1}{N'}\sum _{j'}^{nn} \langle \hat{W_i} \rangle \cdot \langle \hat{W_{j'}} \rangle \right\rangle _{i} \end{aligned}$$

where $\langle \hat{W_i} \rangle$ is the unit vector in the direction of the average outgoing weight for neuron *i*, the domain of the sum *nn* is over the nearest neighborhood of *i*, and $N'$ is the number of adjacent neurons to *i*. Operationally, *O* is calculated by first determining the $\langle \hat{W_i} \rangle$ unit vectors, then the sum of the dot products to all nearest neighbors $j'$ is calculated, and finally this is averaged across the entire network as a measure of the average local order. The maximum value of this parameter is 1 if all unit average weight vectors point in the same direction, and the minimum is 0 for random orientations. To ameliorate edge effects resulting from our open boundary conditions, the neurons at the 2 outermost lattice positions in x and y are excluded from this calculation. Additionally, the z-component of vectors are excluded from this calculation for the same reason. An increase from zero of this parameter as a function of time will be an indication of the formation of synaptic pathways.

To measure the firing activity per neuron of the networks we calculate the firing rate of the whole neuronal population $A(\Delta t_m)$ over a given measurement time period $\Delta t_m = 100$ ms by using,

16 $$\begin{aligned} A(\Delta t_m) = \frac{N_{spikes}(\Delta t_m)}{N_{neurons} \cdot \Delta t_m} \end{aligned}$$

where $N_{spikes}$ is the total number of spikes in that time period normalized by $N_{neurons}$, the total number of neurons in the network.

For the particular case of the central stimulation experiment, we additionally measure the average radial propagation speed of the traveling wave front, *v*, given by,

17 $$\begin{aligned} {v} = \frac{\langle \Delta r \rangle }{\Delta t_{p}} \end{aligned}$$

where $\langle \Delta r\rangle$ is the average of the distances between the center of the stimulation and the location of the firing neurons after a chosen interval of $\Delta t_{p} = 80$ ms following the stimulation event. While this measure takes into account all spikes regardless of their proximity to the wave front, the results are consistent and represent the corresponding traveling wave fronts because the number of spontaneously occurring spikes outside of the propagating wave is proportionally minuscule.

**Fig. 3:**
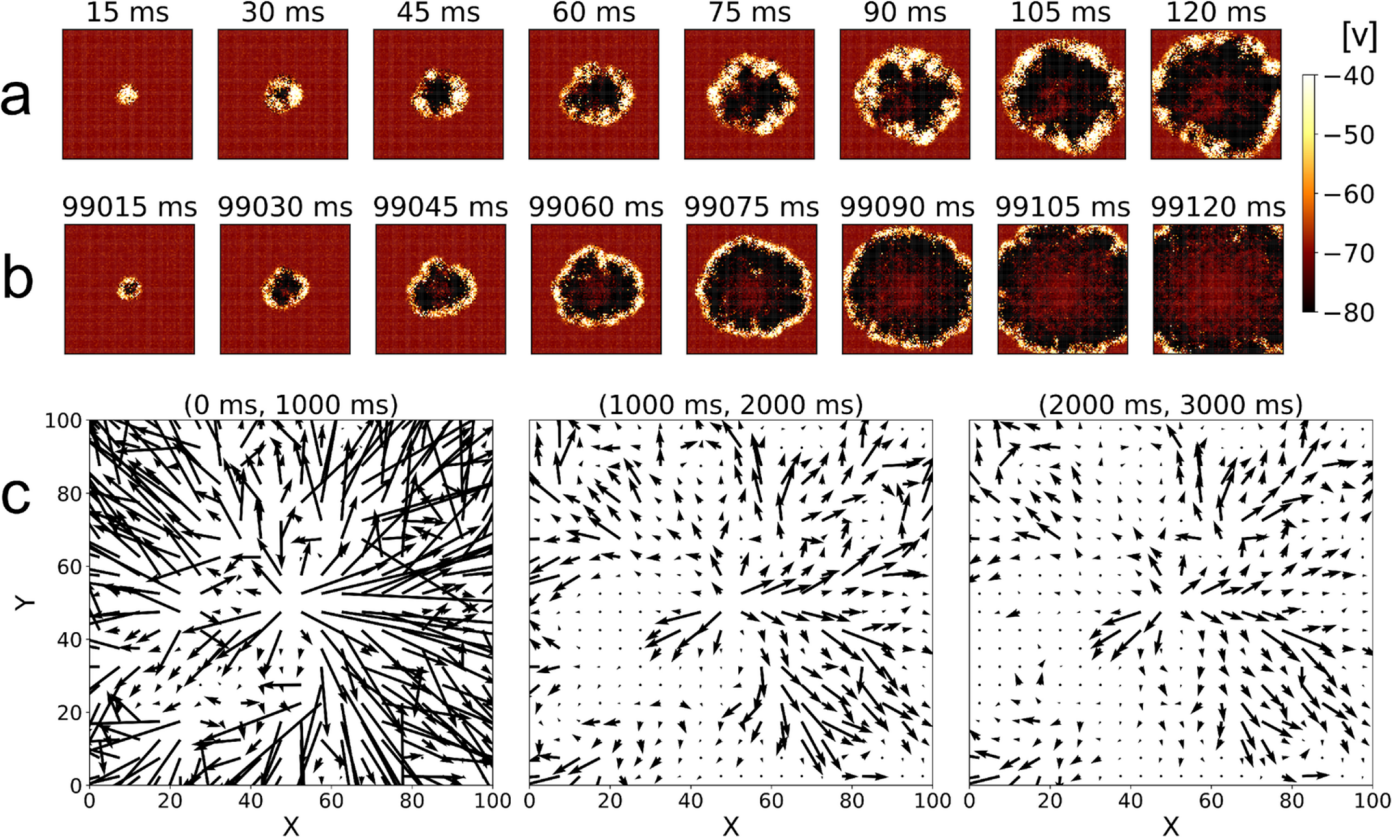
Visualization of network activity after applying central stimulation with $R = 4$ for a single random seed. Successive snapshots show the network activity in 15ms intervals after (a) the first and (b) the hundredth central stimulation, respectively. The color of each (*X*, *Y*) position represents the average membrane potential *v* of the three lattice positions along the *Z* direction. vector fields of the average weight change for each $5\times 5$ region for the first three central stimulations are shown in (c), equivalently scaled for comparison

## Results

### Central stimulation

As described in Section 2.2, the goal of the central stimulation experiment is to determine how travelling waves on networks with different properties affect synaptic structures when the same location is repeatedly activated. Figures 2(a) and (b) show two different snapshots of the network at the same simulation time, without and with plasticity, respectively. These snapshots have in common that there is a spiking front propagating radially outward from the center. However, when STDP is included in Fig. 2(b), there are differences in the distance the wave has traveled in the same time-period after stimulation, as well as in the apparent thickness of the wave. These differences are due to changes in synaptic weights. To visualize the underlying reason for the differences, we show in Figs. 2(c) and (d) the average weight vectors for each $5\times 5$ region throughout the network that show differences in the synaptic structure without and with STDP. Clearly, without plasticity the network remains random as initialized by our model, but with STDP outward synaptic pathways consistent with the direction of the spiking wave front are formed.

To further show how plasticity affects network dynamics as a function of simulation time, Figures 3(a) and (b) show the full progression of one wave starting at different simulation times of the same simulation, for the network with STDP ($R=4$). From the figure it is apparent that the waves propagate outward in both cases, but propagation appears visually faster for the later wave, as demonstrated by the wavefront reaching the edge of the network in a shorter period of time in (b). The main underlying difference is that for the later central stimulation, STDP has had enough time to adjust synaptic weights. To see the timescales at which synaptic weights change, we show in Fig. 3(c) three vector fields of the changes in weights over the first three stimulation periods ($i=1, \; 2, \; 3$).

Of note is that the changes in synaptic weights are larger during the first stimulation as compared to the later ones, suggesting that weights rapidly settle into values that tend to vary less. We point out that the irregularities (non-circularity) of these weight changes, indicated by a lack of perfect radial symmetry, correspond to irregularities in the way that the front propagates outward. This is expected from the random nature of the underlying network (e.g. random distribution of excitatory and inhibitory neurons). Additional examples of network activity with corresponding vector fields of weight changes are included in the SI, Fig. 1.

**Fig. 4:**
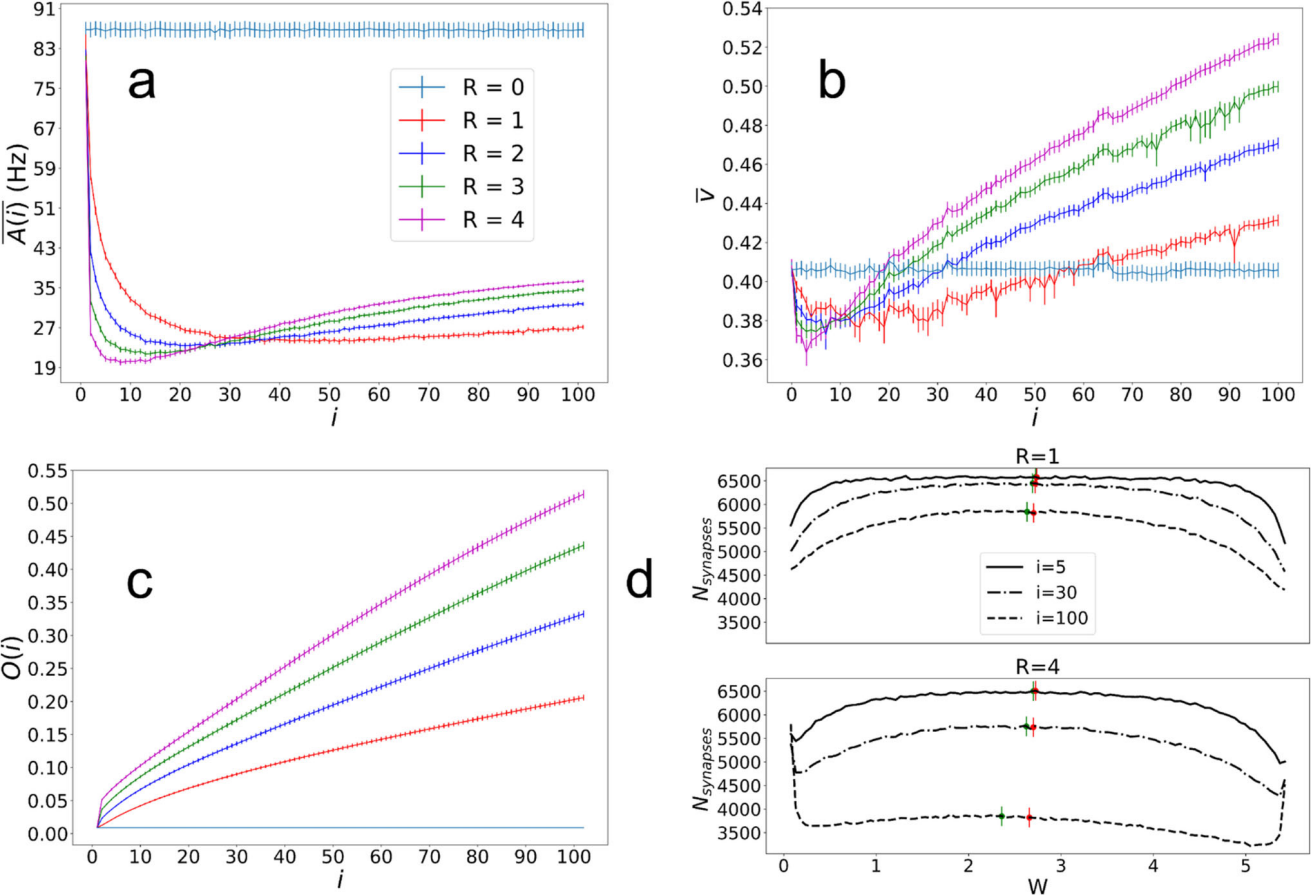
Results from using the central stimulation averaged across 40 random seeds. Error bars represent the standard error of the mean for each measurement. (a) shows the average network firing rate measured over 100 ms after each central stimulation. Colors correspond to different values of *R* as shown in the legend. (b) shows the average propagation speed in the radial direction after each successive central stimulation. (c) shows the local synaptic order parameter as a function of the number of the central stimulation, and (d) shows plots of the distribution of (non-diverged) excitatory synaptic weights for $R=1$ and 4, with 100 bins after stimulation indices $i=5,\;30$, and 100. The red dots represent the median of the non-divergent synapses and the green dots represent the median for all synapses

When examining the average population firing rate per neuron, shown in Fig. 4(a), we find that after the first few stimulations there is a rapid decrease in the firing rate that eventually stabilizes and slowly increases at much later stimulation indices. The position of the minima in $\overline{A}$ as well as its asymptotic value are dependent on the value of *R*. The initial rapid changes indicate a transition from randomly distributed synaptic weights to more ordered synaptic pathways, with the magnitude of the decrease largely due to the asymmetric STDP time-constants ($\tau _-/\tau _+$) that initially favor LTD with $\tau _-$ twice the value of $\tau _+$. When setting equal values to these time constants the magnitude of the decrease is much less (see SI Fig. 2). The reason that the firing rates increase after the minima is that dominating weights at later times are those mostly responsible for LTP. STDP will in turn keep increasing these specific weights until they reach their maximum value, causing the firing rates to eventually plateau.

A similar behavior to $\overline{A(i)}$ is observed in the propagation speed of the spiking wave front, shown in Fig. 4(b), where again the initial ordering and subsequent consolidation of synaptic weights cause a minima to appear. As with $\overline{A(i)}$, the minima is a result of the asymmetric STDP time constants that, when set with symmetric values, the initial decrease in $\overline{v}$ disappears (SI, Fig. 2). As expected, the speed remains constant for the case with no STDP.

To understand trends in the local ordering of the weight vectors, we show in Fig. 4(c) the local order parameter *O* as a function of stimulation index. This figure shows that *O* increases monotonically from zero (corresponding to random networks), indicating increasing local ordering of weights that lead to the formation of synaptic pathways across the network. By definition, an increasing *O* is a measure of increasing directionality in the signal propagation across the network, and consistent with the behavior of the vector fields in Figs. 2 and 3. As a general remark, we note that the parameters $\overline{A(i)}$, $\overline{v}$ and *O*(*i*) will naturally reach limiting values for big enough time due to our prescribed limits on the maximum and minimum values of the synaptic weights.

**Fig. 5:**
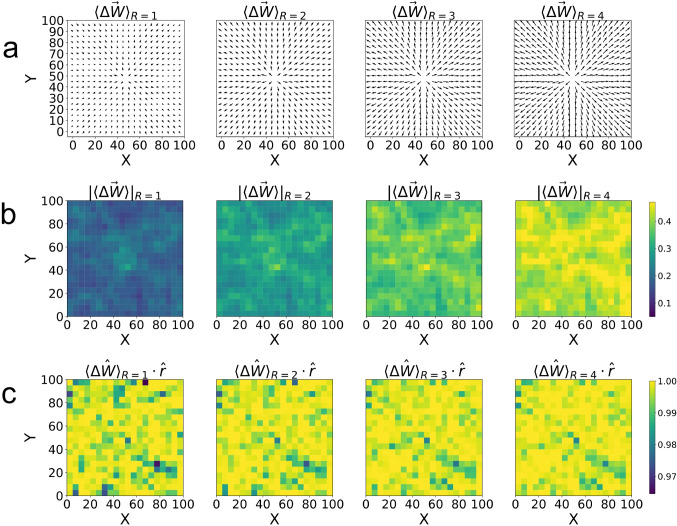
Averaged weight changes across 40 random seeds. (a) shows vector fields of the average weight change for each $5\times 5$ region of the network for $R = 1,\;2,\;3$ and 4. These $5\times 5$ regions are used rather than every neuron position in order to include a sufficient number of synapses in each vector measurement. (b) shows pseudocolor plots representing the magnitudes of the vectors from (a) for each region, and (c) shows pseudocolor plots representing the values of the dot product between the radial unit vector $\hat{r}$ and the unit vector of the average weight change $\langle \Delta \hat{W}\rangle$ for each region

To make sure that our results are not a result of an abnormal distribution of weights as simulation time progresses, in Fig. 4(d) we plot the distributions of weights for non-diverged weights at $i=1,\;30 \; \& \; 100$ for $R = 1,\;4$. Non-diverged weights are those that have not reached either their minimum or maximum allowed values, prescribed in our model. From the figure we can see that while the median synaptic weight for all weights (green dots) shifts to the left, indicating that weights are on average decreasing, the median for non-diverged synapses (red dots) remains more stable. The proportion of total initial synapses that are non-diverged, diverged to maximum, and diverged to zero is included in the SI, Fig. 3, and histograms of excitatory weights without diverged synapses removed is included in the SI, Fig. 4.

To show results from which we can obtain generalizable conclusions, we average data obtained from all of the 40 different realizations. Figure 5(a) shows averaged vector fields of weight changes for $R = 1,2,3,4$ clearly demonstrating that weight change vectors align in the direction of wave propagation. This is because STDP will increase the value of the weights pointing in the direction of propagation, and correspondingly reduce as a function of time the value of the weights that point in the opposite direction. The figure also shows, in a rather natural way, that the magnitudes of these vectors grow with the value of *R*. Figure 5(b) shows that there is uniformity of the magnitude of the weight vectors across the network, while Fig. 5(c) shows uniformity of direction, where the uniform spread of color and their values close to 1 indicate a radial orientation for all cases.

In summary, by using the central stimulation experiment we have demonstrated that the direction of the traveling wave front correlates with the direction of the weight change vectors, and that repeated stimulation in the same direction strengthens synaptic pathways and increases the speed of the waves that propagate through.

**Fig. 6:**
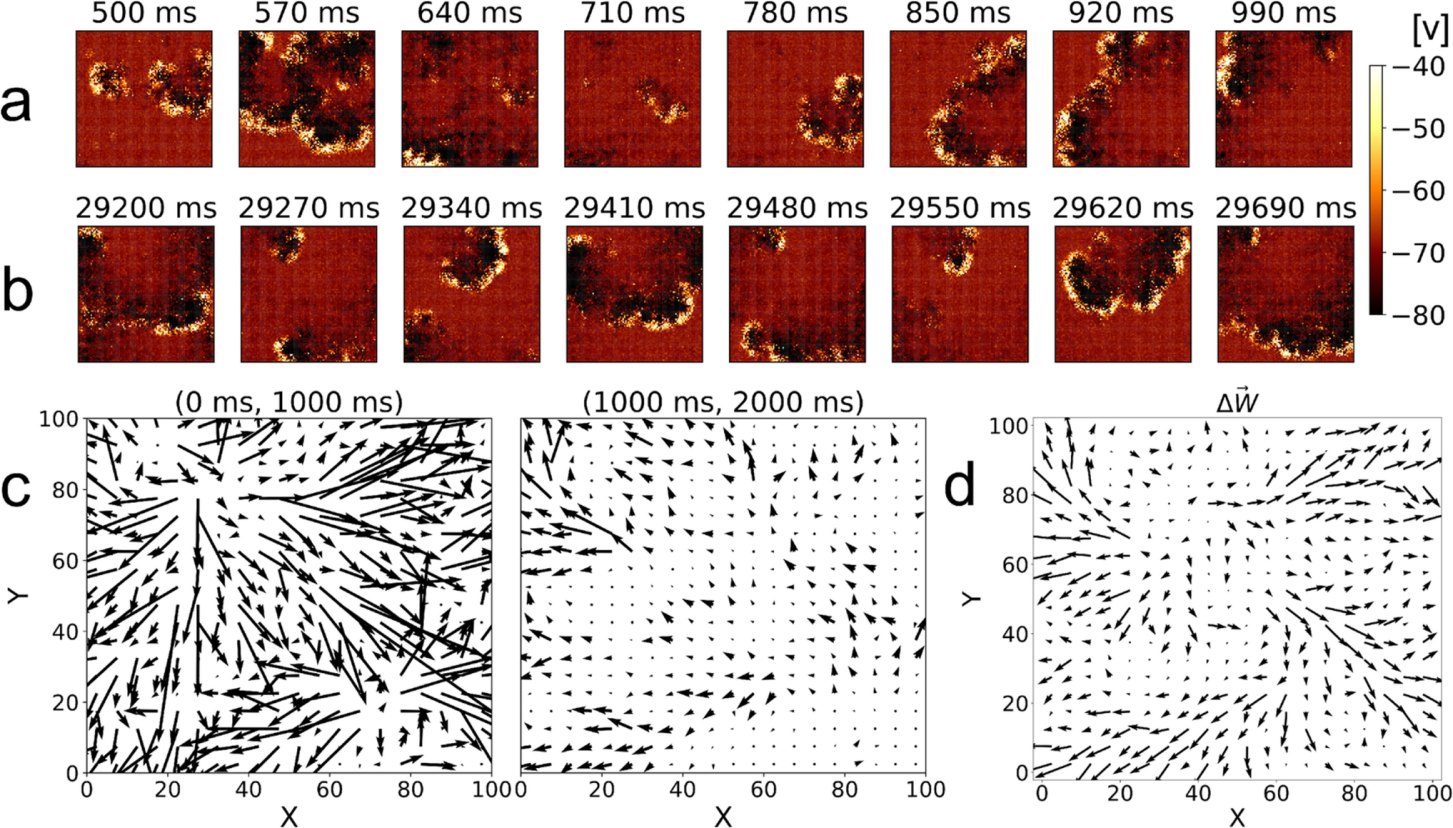
Visualization of network activity for stochastic stimulation with R = 4 for a single random seed. Successive snapshots show stochastic wave activity in 70 ms time intervals starting at (a) 500 ms and (b) 29,200 ms. Each set corresponds to times before (a) and after (b) pathways are formed, respectively. The color of each (*X*, *Y*) position represents the average membrane potential *v* of the three lattice positions along the *Z* direction. (c) shows vector fields of the average weight change for each $5\times 5$ region for the first two 1000 ms periods of simulation, equivalently scaled for comparison. (d) shows corresponding average weight change over the entire simulation

### Stochastic, whole-network stimulation

Now we proceed to investigate whether pathway formation is robust regardless of the origin of wave nucleation within the network using a stochastic stimulation experiment. In Figs. 6(a) and (b) we show time progressions of the stochastic stimulation experiment at different times on the same network. While the weight changes in the top progression have not settled down, shown by the large weight changes in (c), the weight changes and hence synaptic pathways are more stable in the second sequence (b). We note that in (a) there is little evidence of repetitive formation of patterns while in (b) this repetitive behavior is more evident (compare snapshots at 29,200 and $29,410\, ms$) with a period of approximately $200\, ms$, and in this particular example, exhibiting a clockwise wave rotation. The period observed in this figure is dependent on parameters of the model, namely on the strength of connections, time delay parameter, internal parameters of the Izhikevich model neuron, as well as the changing synaptic weights across the network due to STDP. As expected from previous results, the repeating wave patterns are consistent with the vector field of weight changes over the entire simulation shown in Fig. 6(d). Additional examples of wave propagation and corresponding vector fields of weight changes are shown in the SI, Fig. 5.

**Fig. 7:**
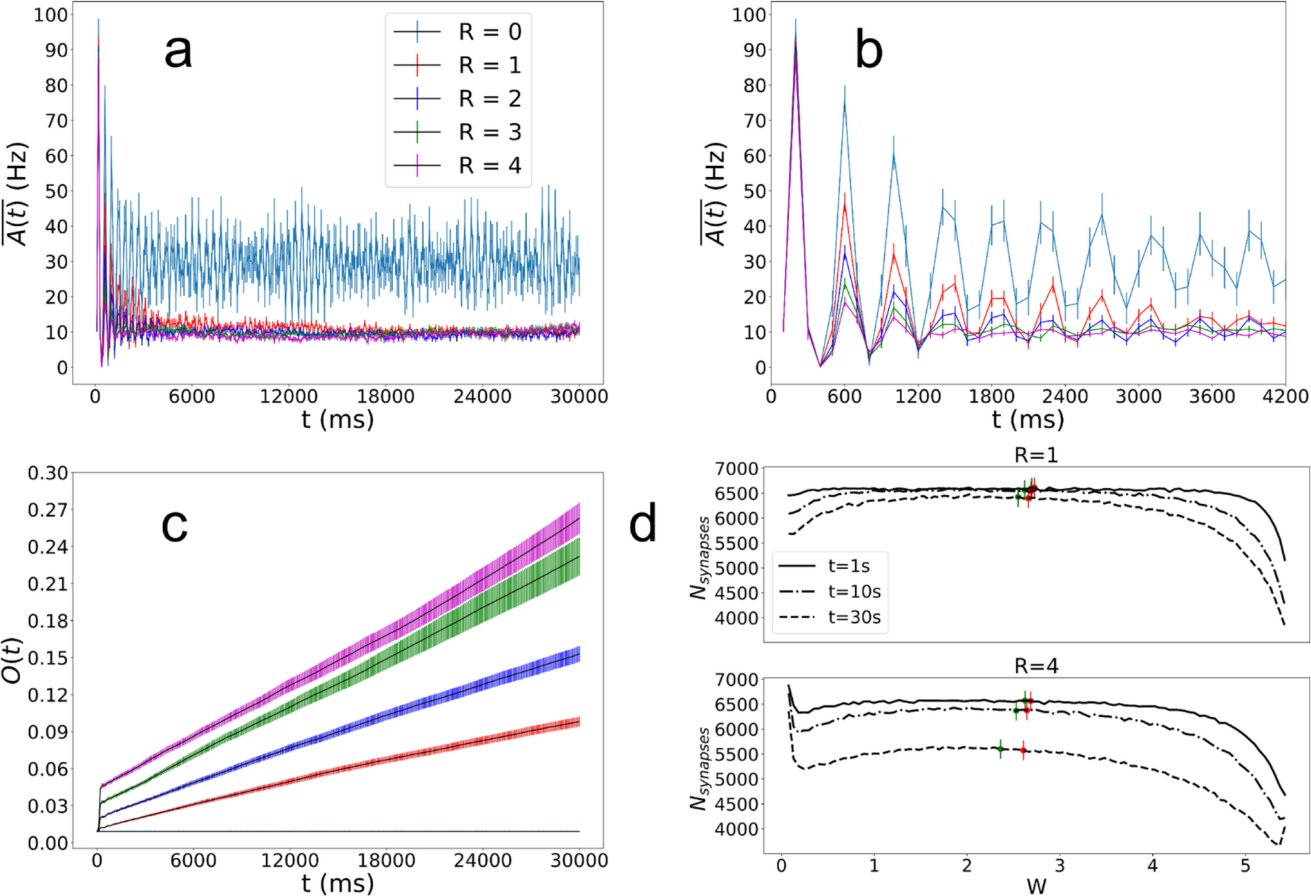
Data from stochastic stimulations averaged across 40 random seeds. Error bars represent the standard error of the mean for each measurement. (a) shows the average network population firing rate averaged over successive 100ms time windows while (b) shows a zoom on the first 4200 ms of (a) to show initial differences in the network population firing rate. (c) shows the local synaptic order parameter, *O*, showing modest, but persistent linear increase. (d) shows plots of the distribution of (non-diverged) excitatory synaptic weights for $R=1$ and 4 with 100 bins at $t=1000,\;10000$, and 30000 ms. Red dots represent the median of the non-diverged synapses and green dots represent the median of all synapses

Similarly to Fig. 3(c), Fig. 6(c) shows the large synaptic adjustments occuring in the first 1000 ms, followed by a more gradual tuning of weights thereafter. Additional examples of this behavior are included in the SI, Figs. 6 & 7.

Figure 7(a) shows the population firing rate, $\overline{A(t)}$, of the network for different *R*. The overall behavior is different than in the case of the central stimulation, where here large fluctuations in $\overline{A(t)}$ result from the different waves propagating in different directions through the network at regular intervals. However, similar to the central stimulation, STDP causes an early attenuation in the population firing rate, $\overline{A(t)}$, of the network, caused by the effects of STDP discussed above. The long-time behavior of $\overline{A(t)}$ is also fundamentally different than in Fig. 3. For all cases with nonzero *R*, firing rates eventually settle to the same value where the value of *R* only seems to affect the time at which this happens, as shown in the zoomed panel in Fig. 7(b). The lack of long-time increase in $\overline{A(t)}$ suggests that weights are exposed to waves that travel in different directions at different times during the repeated cycles.

The local synaptic order parameter *O*(*t*) shows similar behavior to the central stimulation, shown in Fig. 7(c). There is a quick increase in orientational order followed by a gradual monotonic increase for larger simulation times. When comparing with Fig. 4(c) we observe that the absolute values of *O*(*t*) are smaller in the present case, but it is hard to make this direct comparison since the timescales are not identical, one being measured by the index of the stimulation and here by the simulation time.

The distributions of weights shown in Fig. 7(d) show similar behavior as before, with distributions of non-diverged excitatory synapses at $t=1s,\;10s,\;30s$ for $R=1,\;4$. The values for all the medians for all synapses (green dots) decrease, again demonstrating on average a preference for LTD as synapses are adjusted while at the same time not increasing $\overline{A(t)}$ at long times. The median for non-diverged synapses (red dots) is more stable in value. The distribution of excitatory weights without diverged synapses removed is included in the SI, Fig. 4.

In summary, with stochastic stimulation we have demonstrated that unpredictable stimulation throughout the network is still capable of generating traveling waves that, coupled with STDP, can generate permanent synaptic pathways. Early waves are observed to have the greatest effect on the changes of weights of the network, with later activity causing changes to only refine and strengthen the already-formed pathways.

**Fig. 8:**
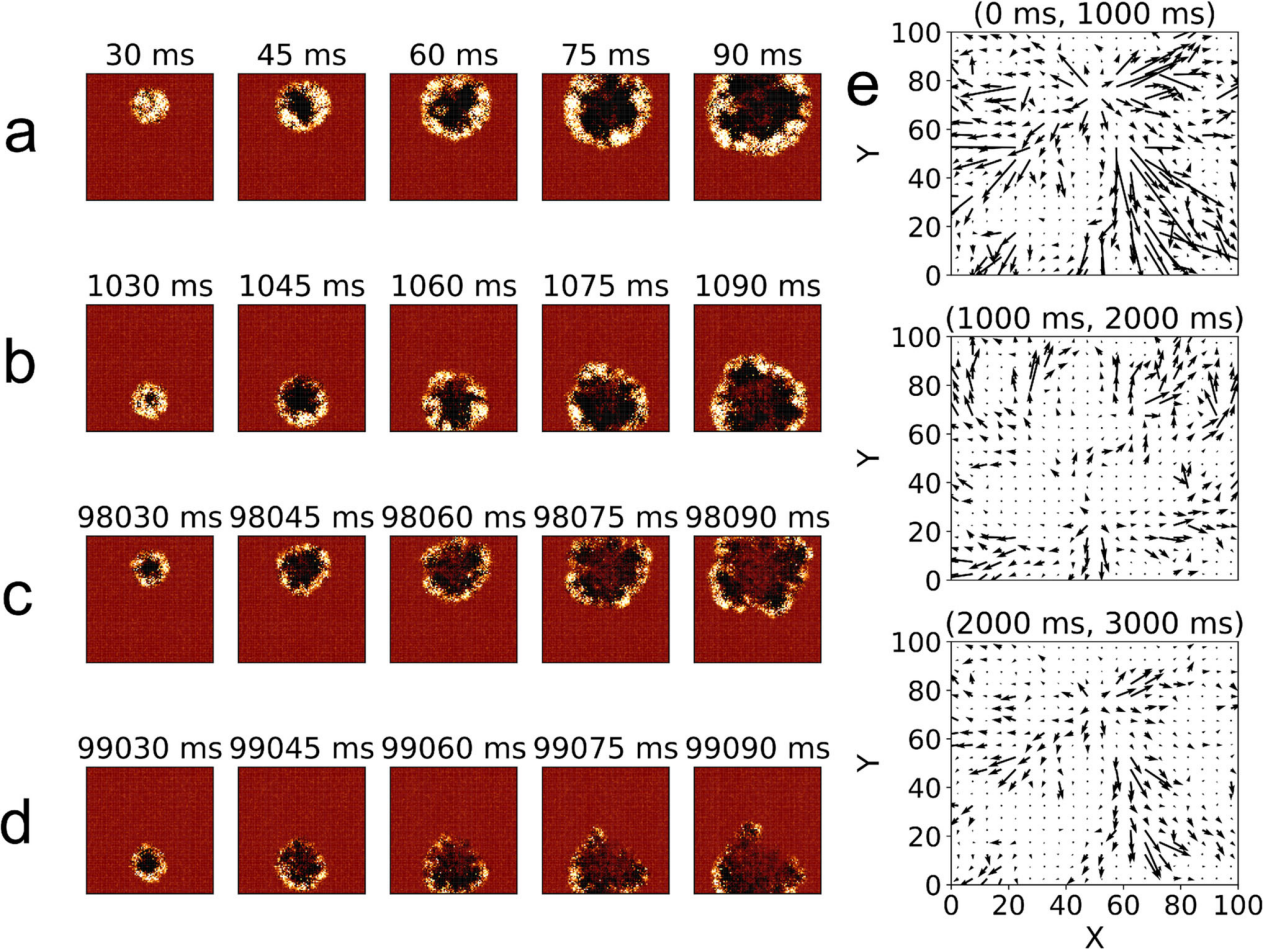
Visualization of network activity for an alternating stimulation with R = 1 for a single random seed. Successive snapshots of network activity at 15*ms* intervals are presented after a burst of stimulation into (a) the top half of the network (initial one), (b) bottom half after 1*s*, (c) top half after 98*s*, and (d) bottom half after 99*s* showing a more muted response. The color of each (*X*, *Y*) position represents the average membrane potential *v* of the three lattice positions along *Z*. The included color scale is equivalent to those in Figs. 2, 3 and 6. (e) shows average vector fields of the weight changes over the first three stimulations, equivalently scaled for comparison

### Alternating stimulation

In the alternating stimulation experiment we now test whether alternating waves with different propagation directions have an effect on the synaptic weights. Although in the stochastic stimulation scenario we presented evidence of alternating waves, here we test, in a controlled and systematic way, the effects of wave alternation. The setup is such that there are alternating stimulations from two locations, one at the top (upper y-axis) and another at the bottom (lower y-axis) of the network every 1000 ms. In this way, the neurons and synapses in the space between the two stimulation sites will be affected by waves traveling upwards or downwards depending on which site has been stimulated. We will see below that this, combined with STDP, will introduce a network-scale competition, resulting in one of the two pathways being strengthened over the other.

Figures 8(a) and (b) show a sequence of snapshots of the first pair of alternating bursts of stimulations while Figs. 8(c) and (d) show the same sequence, but 49 iterations later. By comparing these two sequences, we can see that for the first set of stimulations, both waves behave in a similar way. In contrast, for the later set, the upper traveling wave seems to be similar to the initial one while the lower wave does not, resulting in a more depressed asymmetric wave that seems to be mostly travelling downward. This behavior, on average, seems to potentiate downward synaptic pathways in the space between the sites while upward pathways (in the opposite direction) are depressed. The vector fields shown in Fig. 8(e) demonstrate the relative scale of weight changes in the first three stimulations (top, bottom, top), indicating as in previous experiments, a decreasing effect on the weight changes. In the present experiment, however, this asymmetric decreasing effect has the additional effect that initial weight changes are never fully compensated by the opposing wave, thus establishing an imbalance in the weight changes that continues to develop for the remainder of each simulation. Additional examples of these sequences are included in the SI, Fig. 8.

**Fig. 9:**
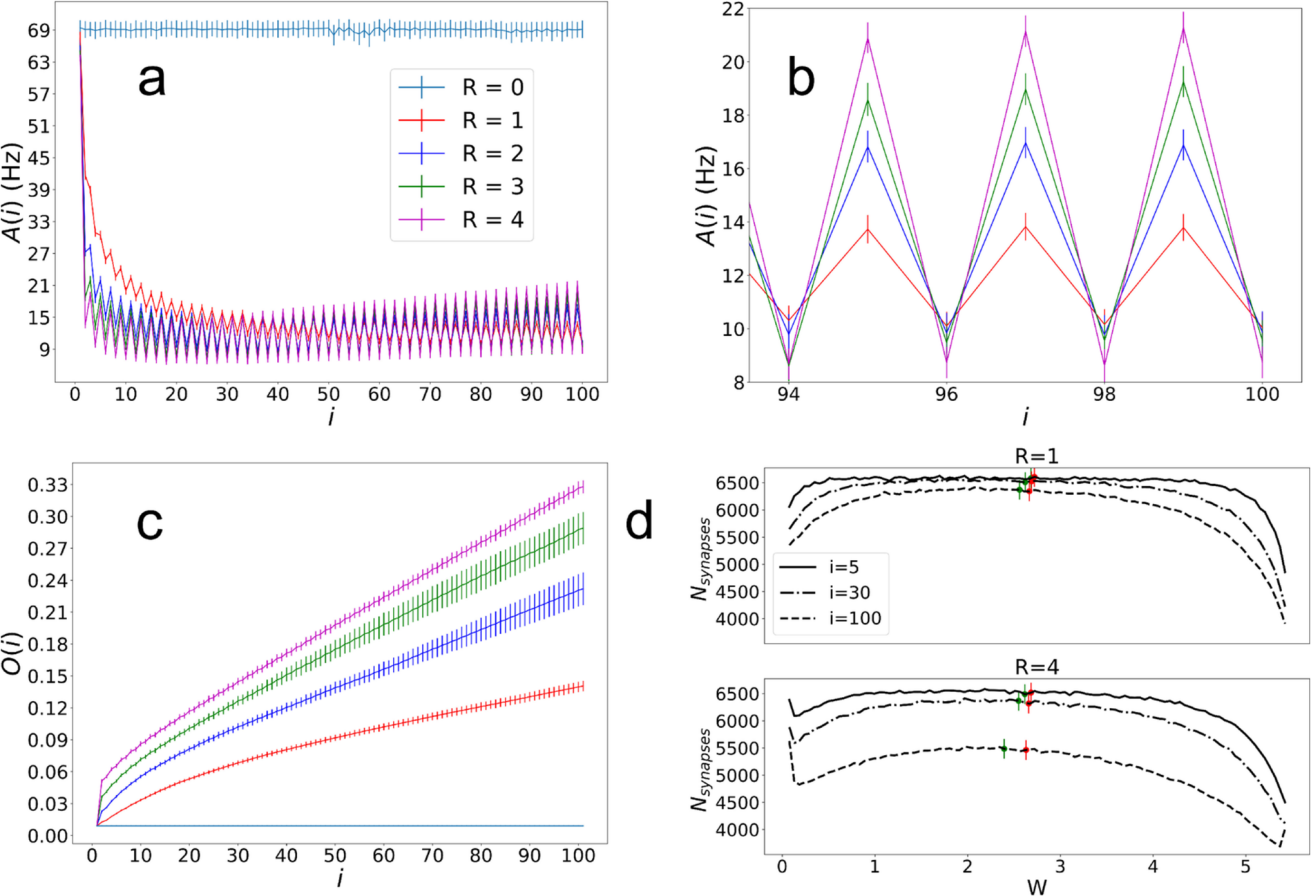
Data from alternating stimulations averaged across 40 random seeds. Error bars represent the standard error of the mean for each measurement. (a) shows the average network firing rates measured over 100*ms* after each stimulation while (b) shows a zoom of the last part of (a) to show individual differences. (c) shows the local synaptic order parameter, *O*, with continuous increases. (d) shows plots of the distribution of (non-diverged) excitatory synaptic weights for $R=1$ and 4 at $i=5$, 30, and 100. Red dots represent the median of non-diverged weights and green dots represent the median of all synapses

Similarly to before, the network population firing rates shown in Figs. 9(a) and (b) show a rapid initial decrease. Different than before, however, is the fact that alternating stimulations show different magnitudes in $\overline{A(t)}$ that are preserved throughout the simulations, where the odd *i* stimulations (top, first) have higher persistent values than the even stimulations (bottom, second). Because the top site is stimulated first, our results suggest that early activity introduces an immediate asymmetry, favoring the result toward the downward pathway between the two sites. Figure 9(b) shows a zoomed view of (a) that shows more clearly the average differences between the upper and lower stimulation sites, including that the alternating rates for different values of *R* are nested within one another where for smaller *R* the difference between the average firing rate in response to the two stimulation locations is on average lower.

Regarding the local order parameter *O*, Fig. 9(c) shows that *O* on average increases with each successive stimulation. In Fig. 9(d) we observe a similar trend to those shown in previous experiments for the distribution of excitatory weights across the networks. The median for all synapses (green dots) decreases, suggesting a dominance of LTD on average, while the median for non-diverged synapses remains relatively stable (red dots). The distribution of excitatory weights without diverged synapses removed is included in the SI, Fig. 4.

**Fig. 10:**
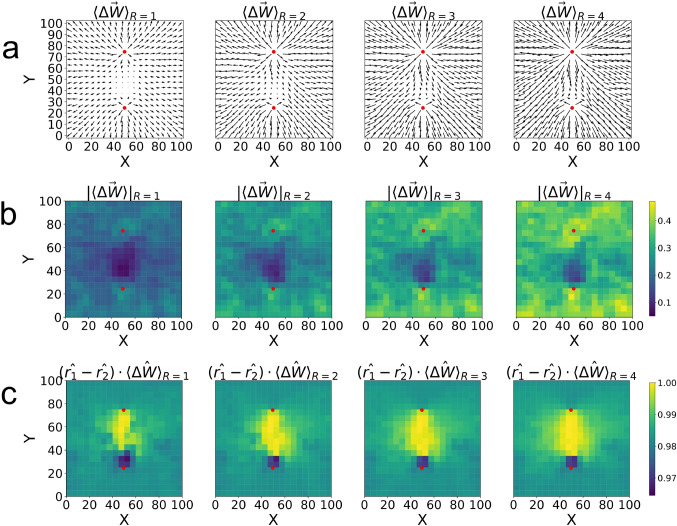
Averaged weight changes across 40 random seeds. Red dots indicate stimulation locations. (a) shows average vector fields of the weight change for each $5\times 5$ region of the network for $R = 1,\;2,\;3$ and 4. These $5\times 5$ regions are used rather than every neuron position in order to include a sufficient number of synapses in each vector measurement. (b) shows heat maps representing the magnitudes of the vectors from (a). (c) shows heat maps representing the dot product between the difference between the higher and lower radial vectors $(\hat{r_1} - \hat{r_2})$ and the unit weight change vector $\langle \Delta \hat{W}\rangle$

Figure 10(a) shows averages of the vector fields of weight changes across all random seeds using the same scale for comparison. These averages show that with higher STDP *R* coefficients, the differences between the downward and upward pathways are more apparent, with the downward pathways showing dominance. In stark contrast with the central stimulation experiment, Fig. 10(b) shows that there is no overall uniformity in the magnitude of the weight change vectors across the network, specifically showing a decreased net change in the space between the stimulation sites, suggesting that the competing alternating waves tend to diminish changes in synaptic weights. However, the changes are not symmetric, as shown by the position of the stimulation centers, where the top is farther away from areas with a decreased average change in weight. In Fig. 10(c) this asymmetry is more evident. In this figure we plot heat maps representing the dot products between the net radial unit vectors $(\hat{r}_1 - \hat{r}_2)$ (as measured from each originating stimulation site) and the unit vectors of the average weight change for each region $\langle \Delta \hat{W}\rangle$. The high values of the heat maps in the region between the two stimulation sites indicate that the vectors of the weight changes align such that they preferentially point downward, toward the bottom stimulation site. This tendency increases for increasing values of *R*. These data show evidence that the first stimulation combined with plasticity permanently influences the shape and form of the pathways, but to be certain we ran an additional experiment where we eliminated the alternating character of the setup in favor of simultaneous stimulation of the top and bottom parts of the network. When doing this we found symmetric results with no preference in the up or down direction for the pathways between the stimulation points (see SI Fig. 9), supporting the idea that plasticity can establish a preference when given an asymmetry in the stimulation.

In sum, the alternating stimulation experiment shows that stimulation asymmetry can introduce competition between pathways that over time results in a net pathway between the two competing sites, with the dominant path dependent on initial conditions and the development of synapses after each stimulation, but more significantly on the initial asymmetry introduced by the first stimulation site. Larger STDP coefficients result in pathways that are more dependent on which site was stimulated first.

## Discussion

Here, we computationally consider waves of neuronal membrane potential activity traveling in quasi two-dimensional networks as a function of synaptic plasticity. In particular, we investigate the extent to which these waves could form robust propagation pathways over time. We consider plasticity by using STDP to adjust the weights of excitatory synapses as waves propagate through the network. We address specific questions by using three specific experiments that differ by their initial conditions and external stimulation to the networks. The questions we address are whether there is a correlation between the propagation direction of traveling waves and the underlying structure of the synaptic weights, whether traveling waves yield stable synaptic pathways, and whether competing waves influence the structure of the network.

For the first question, we use the central stimulation experiment that repeatedly stimulates a small region of the network evoking outward circular traveling fronts. We find that these circular waves strengthen synaptic weights aligned with the direction of propagation while decreasing the weights directed against the direction of propagation. The resulting vector field of weights very closely resemble the traveling waves supported by the network. As a function of the number of stimulations, these outward preferred pathways are strengthened, with accompanying increases in the network population firing rate as well as the speed of outward propagation. For the second question we use the stochastic stimulation experiment in which focal stimulation is applied at random sites throughout the simulations that produce stable pathways with stable population firing rates and increasing local order. Interestingly, although the numerous traveling waves seem to spawn at different sites and traverse in different directions, there tends to emerge a larger-scale pattern with a repetition cycle in the hundreds of milliseconds. Lastly, we investigate competition effects by using the alternating stimulation experiment that, by virtue of the dynamics of STDP, render a preferred synaptic pathway that depends on the initial conditions of the experiment, i.e. on which location is stimulated first.

Previous computational work has already investigated the importance of traveling waves in conjunction with synaptic plasticity. For example, one study investigated a potential mechanism of parallel path planning with the use of spiking wavefronts (Ponulak & Hopfield, 2013). These generated waves traveled through a model hippocampus-like network of place cells, with STDP included to alter synaptic weights as a result of the propagating fronts. The vector fields of weight changes were then used to direct a simulated animal to a desired location within the network. The mechanism of the formation of pathways in this study is similar to that observed in our study, with synaptic pathways forming in the direction of wave propagation. Notable differences, however, are our inclusion of varying neuron types with randomized izhikevich neuron parameters, randomized local connectivity and the inclusion of both inhibitory and excitatory neurons. Our goal in using a more complex network model is to demonstrate robust mechanisms that may be compared directly to biological observations.

In another computational study, researchers investigated the effect of plasticity on feedforward connections from a traveling wave network onto an output neuron (Bennett & Bair, 2015). Their result demonstrated periodic spatiotemporal patterns of increasing and decreasing synaptic output, but they did not focus on synapses within the traveling wave network itself, as is the focus in the present study. A more recent study specifically demonstrated the formation of ‘poly-synaptic’ pathways using STDP and leaky integrate and fire neurons (Ito & Toyoizumi, 2021). This paper, however, utilized a reward-dependent STDP model with synapses that continuously change based on a dopaminergic reward signal and an STDP eligibility trace rather than discrete weight changes after individual pairs of pre and postsynaptic spikes. Their model also included rate-controlled excitatory neurons and a custom wave-propagation model rather than propagation via synaptic connections alone. By comparison, our model uses built-in synapses with distance-dependent time-delays to model the biological mechanisms of wave propagation in neuronal circuits, and unmodified excitatory STDP to demonstrate resultant changes in synaptic connectivity.

Some aspects of our work have connections with experiments. For example, our results show that on average, synaptic weights decrease with the passing of a well-formed travelling wave across the network. In observational studies, data suggests that slow-wave propagation (1-2 Hz) during deep sleep may act as a mediator for plasticity processes (Massimini et al., 2009). Evidence suggests that propagating slow-wave signals may be responsible for increased LTD during sleep, decreasing the synaptic strength of connections that were over-potentiated during wakefulness (Tononi & Cirelli, 2006, 2014) referred to as the Synaptic Homeostasis Hypothesis (SHY). Comparison could be drawn between this effect and the tendency of LTD to dominate in our networks, most evidently during the early times in our simulation, which causes attenuation in the network population rate in response to stimulus in early simulation.

Additionally, evidence links the propagation of signals during development to the formation and organization of networks in the visual system (Shatz, 1996). The formation of pathways in an initially random network, as we study here, could be compared to this organization of neuronal pathways during early development. Further, while the initial development of networks are a result of intrinsic biological mechanisms and spontaneously evoked waves, refinement of these networks eventually become dependent on experience-dependent neuronal activity, often taking the form of propagating waves (Zhang & Poo, 2001). If traveling waves play a role in the formation and refinement of neuronal circuits during development, the measured formation of pathways in our study may approximate a mechanism of those synaptic refinements.

A plausible experimental setup to test some of our results could involve pairing measurements of the speed of observed traveling waves with local observations of potentiation and depression in individual synapses. This approach would allow for the direct observation of how changes in synaptic efficacy vary as a result of traveling waves that pass through. Examples of the feasibility of such measurements are given by speed measurements shown in previous work using Magnetoencephalography (Zhigalov & Jensen, 2023) and the use of electrode arrays to measure the Local Field Potential (Rubino et al., 2006). Measurements of synaptic modifications from multiple directions could be used to determine whether LTP occurs primarily in the direction of wave propagation along with observable changes in speed, as suggested by our computational results.

Our current model has several limitations. One of them is our use of open boundary conditions, which not only affects the size of the network, and by direct inference, the size of tissue that our modeling would represent, but also introduces the small but non negligible fact that edge neurons have fewer total connections. This, in principle does not substantially affect our results being only a surface effect. Another shortcoming of our model is the necessary consideration of a hard maximum for synaptic weights that results in the effects of LTP suddenly halting once a neuronal synapse reaches this maximum. However, increasing the value of the maximum weight does not change the overall conclusions of our study (see SI Fig. 10), but do show differences in the rate at which $\overline{v}$ and $\overline{A(i)}$ eventually increase. Additionally, changing the initial range of weights by using different values of *K* does not significantly alter the trends in the average wave speed or local order parameter (see SI Fig. 11). Further, our model is limited to STDP and does not take into account many other additional learning rules that reflect differences in plasticity for differences in neuron types, including for inhibitory synapses (Capogna et al., 2021), which remained constant in our simulations, as well as the plasticity observed in synaptic time-delays (Lin & Faber, 2002).

In sum, here we have presented a biologically-inspired computational model that demonstrates the pathway forming and strengthening capabilities of traveling waves with plasticity as a possible mechanism for the development of network architecture. However, more thorough research is required to further investigate the full interaction between plasticity and traveling waves in real biological systems and their computational and cognitive role. More experiments are needed geared towards the study of the formation of cortical networks during development and the influence of traveling waves during this time period for which our results point to marked changes in network dynamics. Microscopic studies on the synaptic modification of local synapses in a system where traveling waves are observed would also be effective in testing the results of this study. In addition, more computational experiments are needed to further investigate the formation of pathways under synaptic plasticity using additional biologically inspired neuronal networks and more complex synaptic learning rules to help determine the robustness of this phenomenon. Continued research in this area will allow further progression in the endeavor to better understand learning, development, and sensory processing as it occurs within the brain.

## Supplementary Information

Below is the link to the electronic supplementary material.

Supplementary file 1 (zip 129944 KB)

## Data Availability

No datasets were generated or analysed during the current study.

## Footnotes

1 Code and instructions for recreation of our results using the BRIAN Neural Simulator are available through our Github repository at: https://github.com/kjb-research/JCN-Traveling-Wave-Pathways.
