# Supplementary material for: Neuronal traveling waves form preferred pathways using synaptic plasticity
Source: J Comput Neurosci. 2024 Dec 27;53(1):181–98. doi: 10.1007/s10827-024-00890-2 (PMC11868204; doi:10.1007/s10827-024-00890-2)
Supplement: Supplementary file 1 — (zip 129944 KB) [file 10827_2024_890_MOESM1_ESM.zip › Supplementary_Information.pdf]

# Supplementary Information: Neuronal Traveling Waves Form Preferred Pathways Using Synaptic Plasticity

Kendall Butler[1] and Luis Cruz[1*]

[1]Department of Physics, Drexel University, 3141 Chestnut Street, Philadelphia, 19104, PA, USA.

*Corresponding author E-mail: ccruz@drexel.edu;
First author: kjb388@drexel.edu;

**Published via: Journal of Computational Neuroscience (Springer)**

This document includes additional examples and tests used to further support the processes and conclusions discussed in the main text. Each figure here is referenced in the main text as, for example, 'SI Figure 1' for Figure 1 of this document.

Supplementary movies have been separately provided. The names of the files directly correspond to the figures of the manuscript or the SI.

Jump to: SI Figure 1, 2, 3, 4, 5, 6, 7, 8, 9, 10, 11

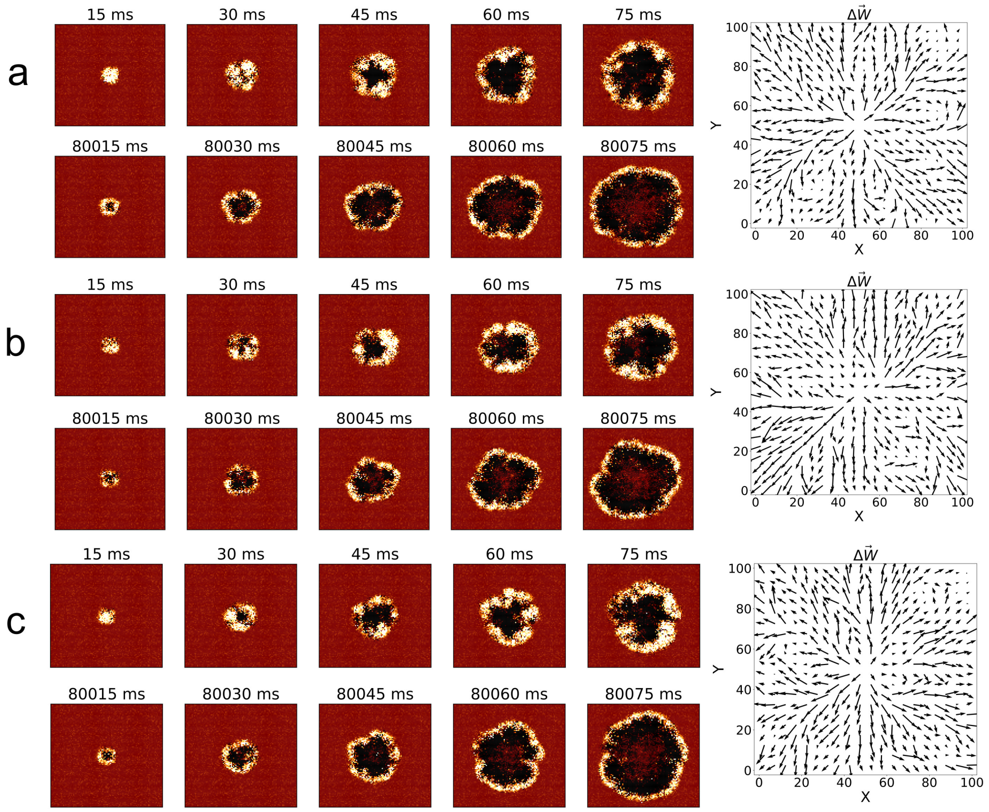


**Fig. 1**: (a)-(c) Three additional examples of the central stimulation, each using a different random seed. For each, the top row shows network activity in the early simulation while the bottom row shows network activity for late simulation times($> 80s$). Snapshots of the voltage map are shown for in 15 ms intervals. To the right, we show the corresponding vectormaps of weight changes across the network.

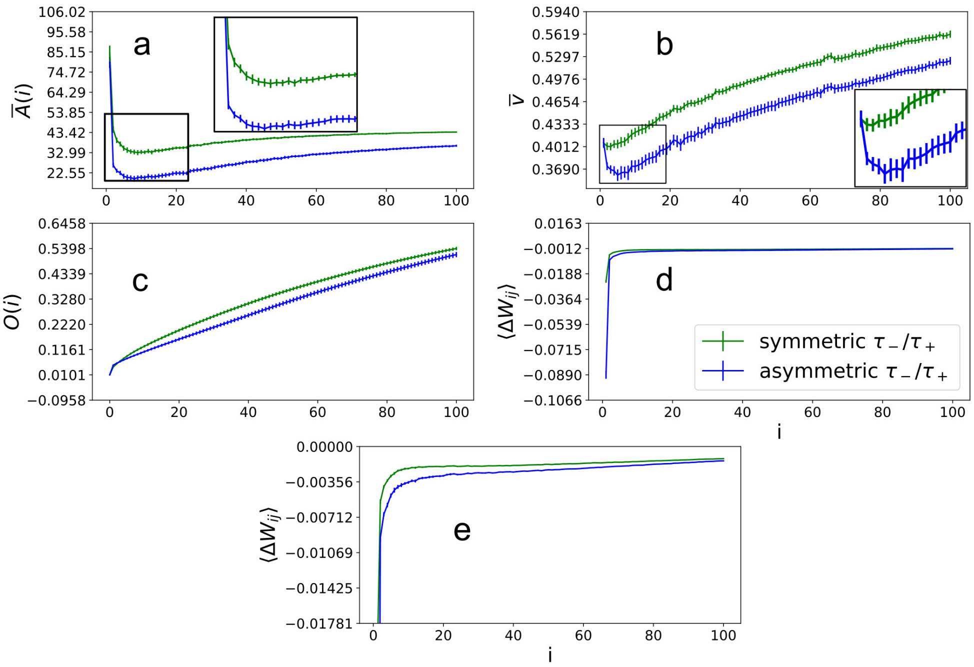


**Fig. 2**: Comparison between data with asymmetric $\tau_-/\tau_+$ (as used in the main text) and symmetric ($\tau_- = \tau_+ = 16ms$) over 10 random seeds for central stimulation. (a) Population rates still drop, but to a lesser extent for symmetric time-constants. (b) The initial drop in speed is removed with symmetric time-constants. (c) The local order parameter increases similarly in both cases. (d) The average of the change in weight for every excitatory synapse favors LTD, but to a lesser extent overall for symmetric time constants. (e) Zoom on the average change in weight for every excitatory synapse (from e) for clarity.

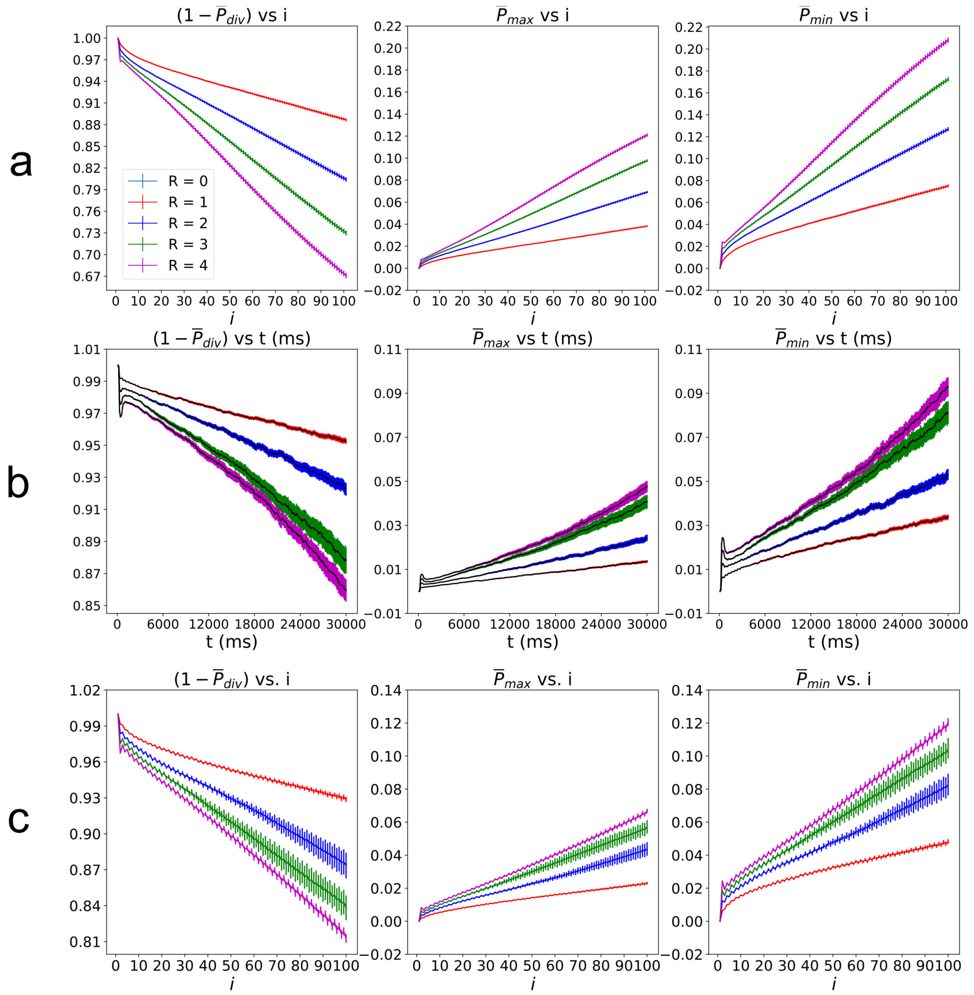


**Fig. 3**: (a) Proportion of divergence of synapses for the central stimulation. From left to right - the synapses that have not diverged, the proportion diverged to maximum and the proportion diverged to zero. (b) Proportion of divergence of synapses for the stochastic stimulation. (c) Proportion of divergence of synapses for the alternating stimulation.

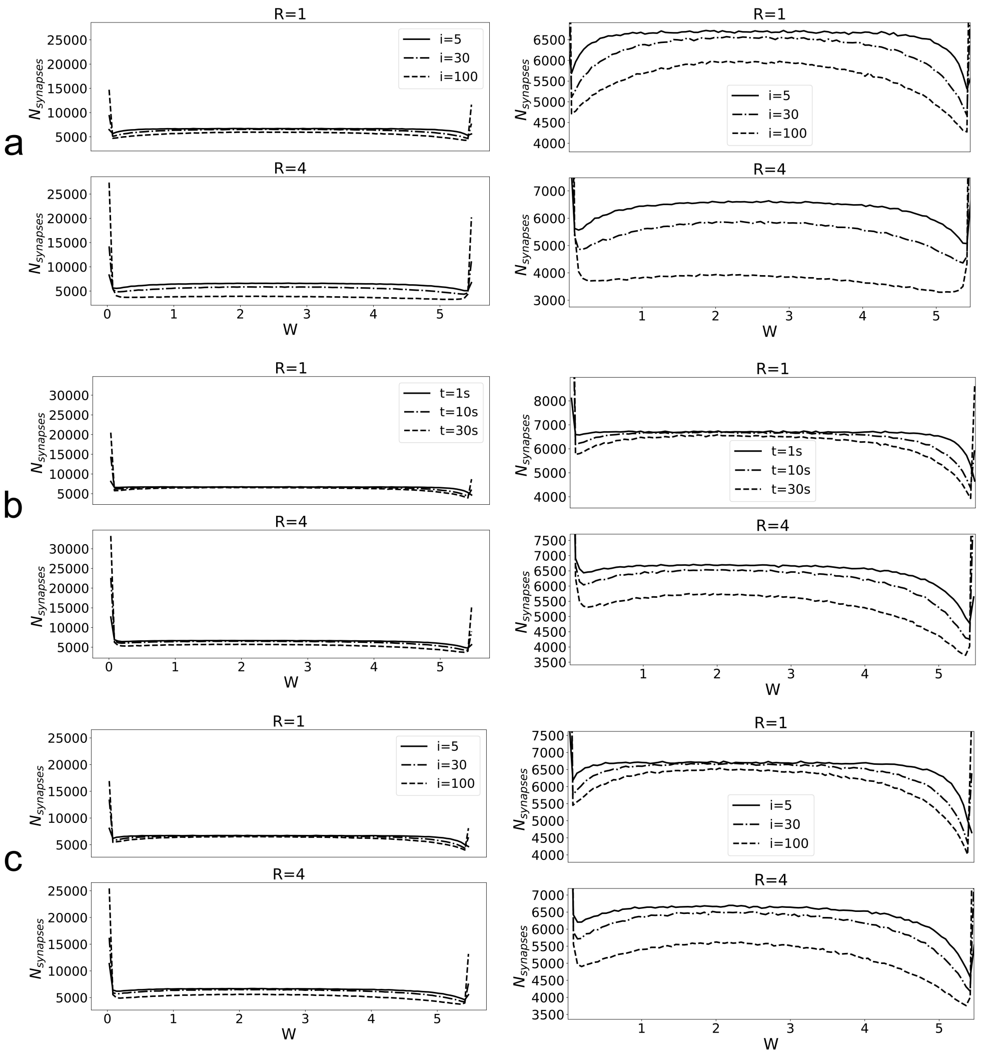


**Fig. 4**: Histograms of weight distributions including diverged synapses shown in the same way as in the main text. These include the full (left) and zoomed (right) distributions where (a) through (c) correspond to the central stimulation, the stochastic stimulation, and the alternating stimulation.

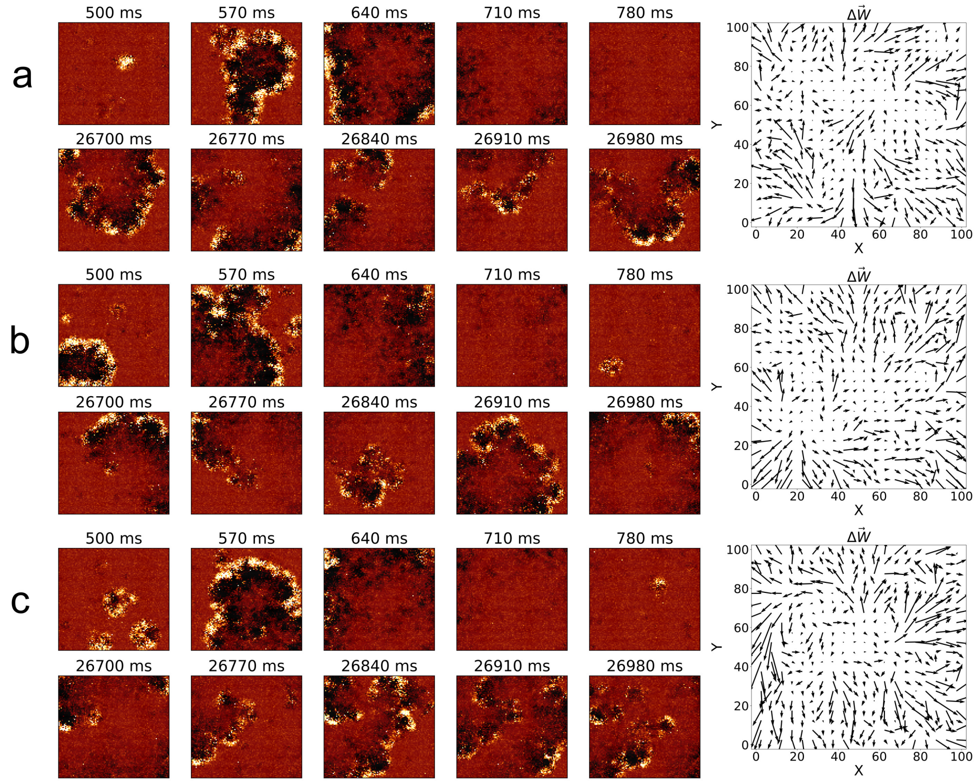


**Fig. 5**: (a)-(c) Three additional examples of the stochastic stimulation with $R = 4$ for three different random seeds. Snapshots of the voltage maps are shown in 70 ms intervals. To the right, we show the corresponding vectormaps of weight changes across the network for each specific realization.

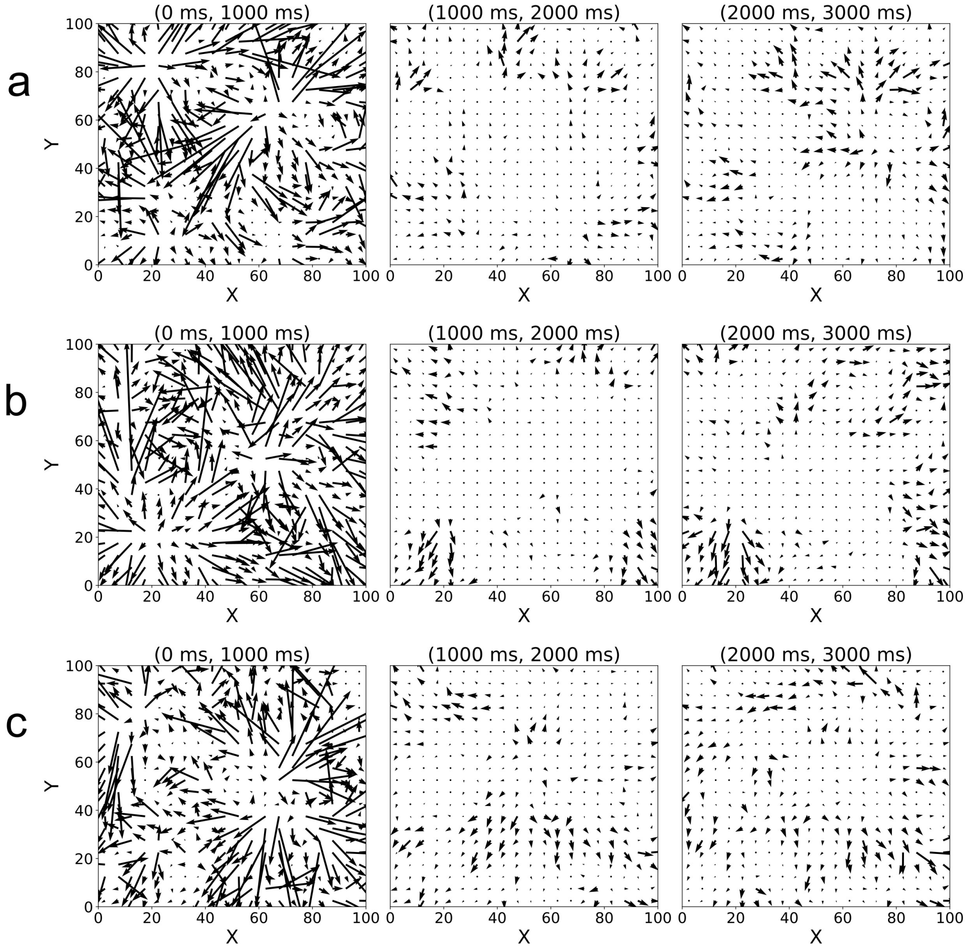


**Fig. 6**: (a)-(c) Three additional examples of vectormaps of weight changes for the stochastic stimulation with R = 4 during the first three 1000 ms-long intervals of simulation time. For all the examples, the greatest change in weights occurs at the early simulation period, followed by a more gradual synaptic adjustments over time.

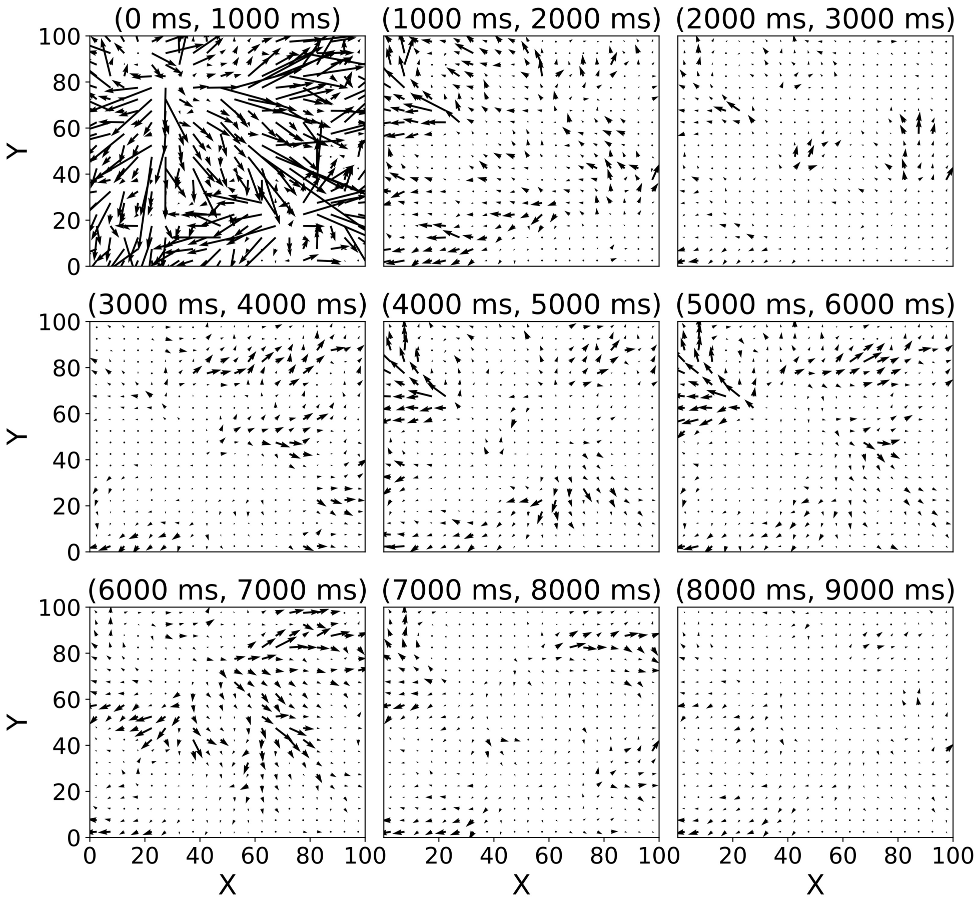


**Fig. 7**: Extended example of 1000 ms-long intervals of weight changes, scaled for the stochastic stimulation up to a time of $9s$. Changes in weights can clearly be seen decreasing in magnitude as the simulation progresses.

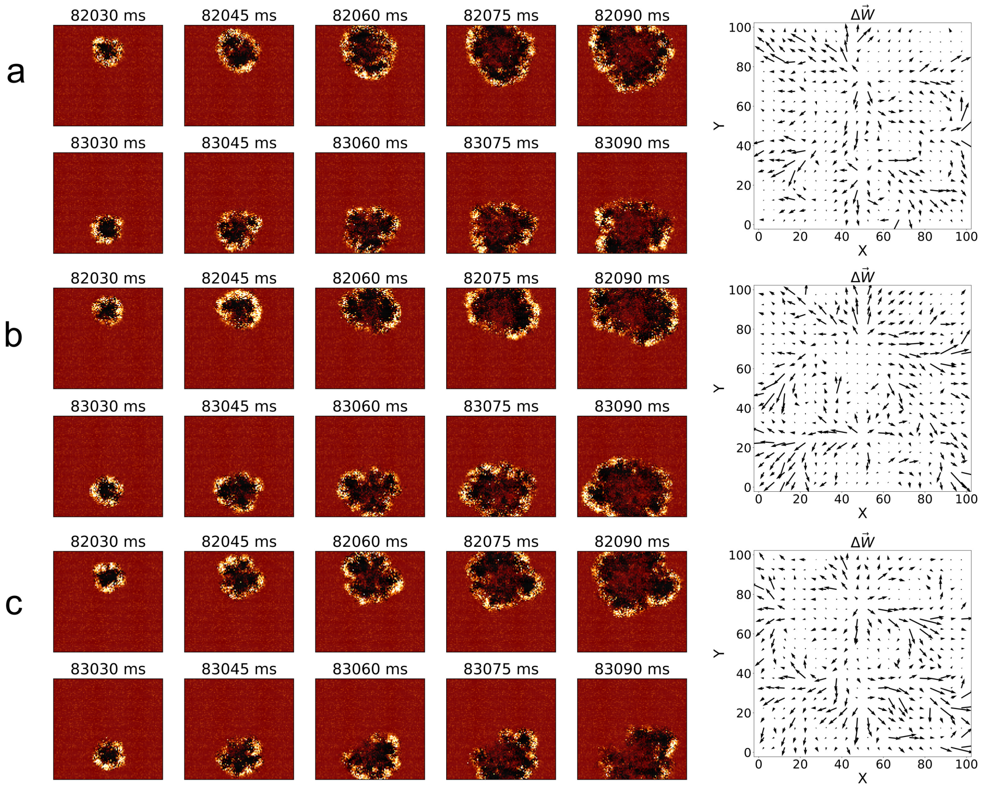


**Fig. 8**: (a)-(c) Three additional examples of the alternating stimulation $R = 1$ for three different random seeds. For each, the top row shows network activity in the early simulation while the bottom row shows network activity in the late simulation. Snapshots of the voltage maps are included in 70 ms intervals. To the right, the corresponding vectormaps of weight changes across the network are shown for each specific random seed.

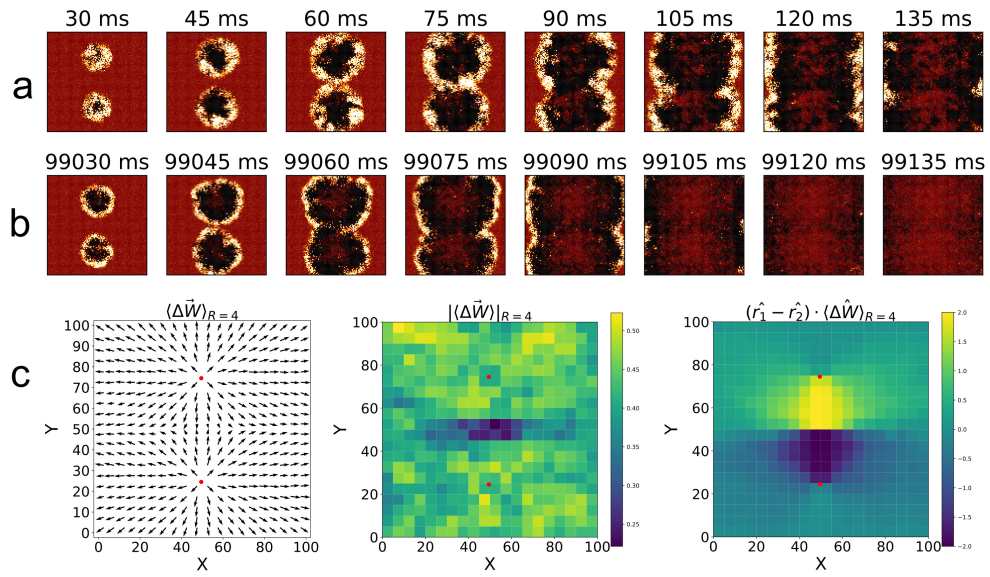


**Fig. 9**: Simultaneous stimulation experiment, to be directly compared to the alternating stimulation experiment of the main text. Here, the top (centered on {49.5,74.5} and bottom (centered on {49.5,24.5}) $8 \times 8 \times 3$ regions of the network receive simultaneous stimulation every 1000 ms using $R = 4$. (a) shows the network activity after the 1st stimulation and (b) shows the network activity after the 100th stimulation. (c) shows, from left to right, the vectormap of the weight change, a heat map representing the magnitude of the vectormap, and a heat map representing the dot product between $(\hat{r}_1 - \hat{r}_2)$ and $\langle \Delta \hat{W} \rangle$.

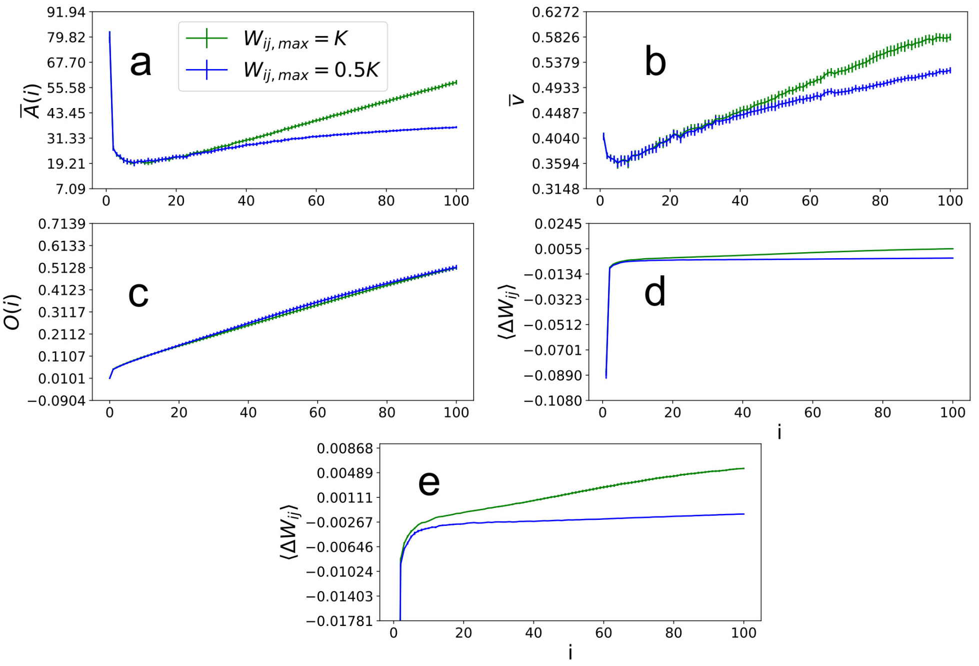


**Fig. 10**: Comparison between various results using as maximum weight $W_{ij,max} = 0.5K$ and $W_{ij,max} = K$ for the central stimulation. (a)-(d) show the population firing rates, average speed, local order parameter, and average weight change, respectively. (e) is a zoom of the average weight change in (d) for clarity. A larger maximum weight of $W_{ij,max} = K$ only causes the firing rates and velocities to increase at a greater rate as time progresses. Values and error bars are calculated using 10 random seeds.

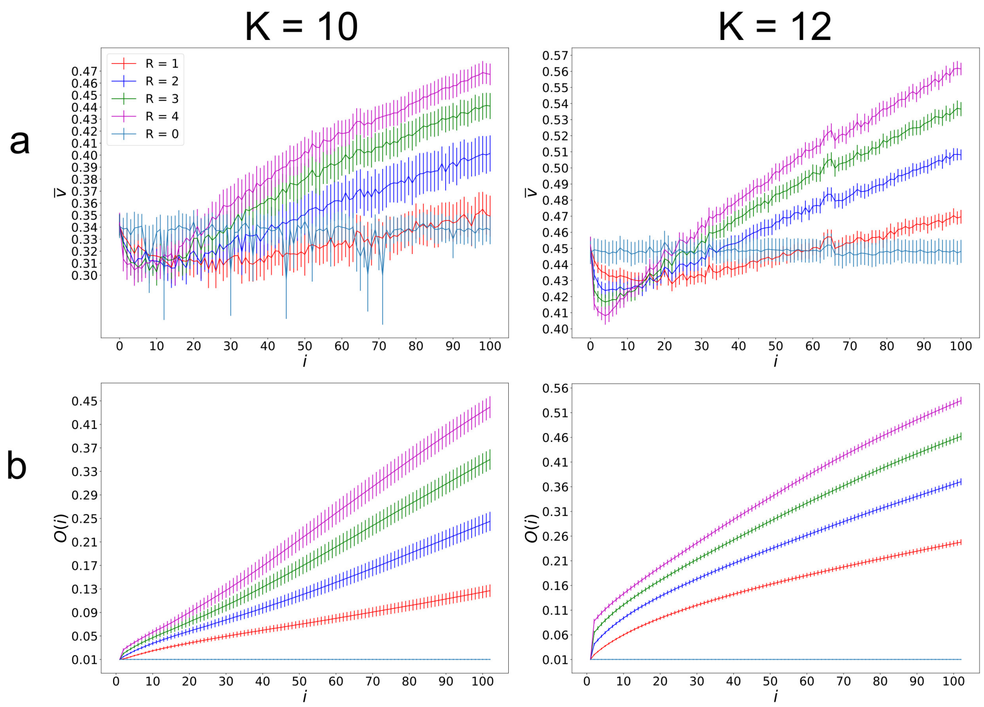


**Fig. 11**: Average speed (a) and local order parameter (b) using $K = 10$ and $K = 12$ for the central stimulation experiment. Values and error bars are calculated using 10 random seeds.
